# Screening and Selection of Drought-Tolerant High-Yielding Chickpea Genotypes Based on Physio-Biochemical Selection Indices and Yield Trials

**DOI:** 10.3390/life13061405

**Published:** 2023-06-17

**Authors:** Prakash N. Tiwari, Sharad Tiwari, Swapnil Sapre, Anita Babbar, Niraj Tripathi, Sushma Tiwari, Manoj Kumar Tripathi

**Affiliations:** 1 Biotechnology Centre, Jawaharlal Nehru Krishi Vishwa Vidyalaya, Jabalpur 482004, India; tiwarisprakashn051194@gmail.com (P.N.T.); swapnil.spr@gmail.com (S.S.); 2 Department of Plant Breeding and Genetics, Jawaharlal Nehru Krishi Vishwa Vidyalaya, Jabalpur 482004, India; anitababbarjnkvv@gmail.com; 3 Directorate of Research, Jawaharlal Nehru Krishi Vishwa Vidyalaya, Jabalpur 482004, India; nirajtripathi@jnkvv.org; 4 Department of Plant Molecular Biology & Biotechnology, Rajmata Vijayaraje Scindia Krishi Vishwa Vidyalaya, Gwalior 474002, India; sushma2540@gmail.com

**Keywords:** chickpea, drought stress, selection indices, drought tolerant genotypes

## Abstract

Chickpea production is seriously hampered by drought stress, which could be a great threat in the future for food security in developing countries. The present investigation aimed to screen the drought-tolerant response of forty desi chickpea genotypes against drought stress through various physio-biochemical selection indices and yield-attributing traits. Principle component-based biplot analysis recognized PG205, JG2016-44, JG63, and JG24 as tolerant genotypes based on physiological selection indices. These genotypes retained higher relative water content, stomatal conductance, internal CO_2_ concentration, and photosynthetic rate. ICC4958, JG11, JAKI9218, JG16, JG63, and PG205 were selected as tolerant genotypes based on biochemical selection indices. These genotypes sustained higher chlorophyll, sugar and proline content with enhanced antioxidant enzyme activities. With respect to yield trials, JAKI9218, JG11, JG16, and ICC4958 had higher seed yield per plant, numbers of pods, and biological yield per plant. Finally, JG11, JAKI9218, ICC4958, JG16, JG63, and PG205 were selected as tolerant genotypes based on cumulative physio-biochemical selection indices and yield response. These identified drought-tolerant genotypes may be further employed in climate-smart chickpea breeding programs for sustainable production under a changing climate scenario.

## 1. Introduction

Legumes play a significant role in human diet because they not only complement the nutrients in a cereal diet but also improve the taste and texture of staple dishes [1,2]. Chickpea is a nutrition-rich grain legume and serves as an inexpensive source of high-quality daily protein as compared to animal protein, so is vital for nutritional security in developing countries, especially the vegetarian people of India [3,4]. It also serves as an enhancer of soil fertility through biological nitrogen fixation and fits in various crop rotation systems for the improvement of soil fertility [5,6]. It is also known as Bengal Gram or Garbanzo, and originated from Turkey [7]. It is ranked third after dry beans and peas worldwide [8,9]. Globally, chickpea occupies 14.8 Mha area, spanning over 59 countries, with an annual production of 15.1 million tons [10]. The major global production of chickpea comes from Asian countries; India shares 70% of the global chickpea area and 67% global chickpea production as the largest chickpea-producing country, followed by Pakistan, Turkey, Australia and Myanmar [11]. Based on seed morphological traits, chickpea is separated into two groups, i.e., desi type with microsperma and Kabuli type with macrosperma [12,13]. Desi type is more important than Kabuli type, as it covers approximately 80–85% of global chickpea production [14]. Desi chickpea is a potential source of nutritional components, i.e., high-quality proteins composed of albumins and globulins in large quantities, amino acids, essential fatty acids, trace elements and minerals [15]. 

Chickpea is frequently grown as rainfed crop in arid and semiarid regions, where water requirement is mainly received with either seasonal rainfall or stored moisture under soil [16,17]. In the last few years, unpredicted climatic changes resulting in high temperature (heat stress) and unusual rainfall (floods) and drought stresses are becoming major threats for crop production [18,19,20,21,22]. Among climatic changes, low moisture and high temperature stresses are the most important yield-limiting stresses in chickpea [23]. Chickpea is most sensitive to water stress at pre-flowering and early pod filling stages [1,4]. It is estimated that terminal drought alone can cause up to 50% of yield losses in chickpea [4,24]. 

Genetic improvement could be a less expensive and more long-lasting solution for better drought adaptation in chickpea than agronomic options. However, an understanding of yield maintenance under low water supplies becomes increasingly difficult because of several mechanisms employed by plants for maintaining growth and development [25]. To experience better stability of grain yield under drought, trait-based breeding strategies are being increasingly emphasized above yield-based breeding because grain yield is greatly affected by genotype × environment interactions and depicts low heritability [26]. Trait-based breeding also enhances the probability of crosses, which result in additive gene action under drought conditions. 

For chickpea breeders, the breeding of drought-tolerant cultivars has been a tough task because of the unavailability of good selection indices. The lack of genetic divergence and a good source of resistance/tolerance to different abiotic stresses has been a major obstacle in the development of high-yielding drought-tolerant chickpea cultivars [27]. The screening and selection of chickpea germplasm line (s) based on diverse morpho-physiological and biochemical traits becomes a pre-requisite for crop improvement under drought stress [26]. Although similar efforts have been made with a major focus on morpho-physiological and biochemical traits contributing to drought tolerance in chickpea [4,16,26,28], limited detail about the terminal stage drought tolerance of the same genetic material are available. Thus, to fill this gap, the present investigation was conducted to assess the effect of terminal drought stress in chickpea genotypes by evaluating key drought-tolerant indicator traits and to select high-yielding drought-tolerant chickpea genotypes, especially those cultivated in India.

## 2. Materials and Methods 

The experiment was performed in a randomized completely block design (RCBD) with three replications during the post-rainy seasons of 2020–2021 and 2021–2022 under a rainout shelter at Biotechnology Centre, Jawaharlal Nehru Krishi Vishwa Vidyalaya (JNKVV), Jabalpur (23°10′ N 79°59′ E). To study the effect of normal irrigated and terminal drought-stressed conditions on the morpho-phenology, physiology, biochemistry, yield and other traits of desi chickpea at reproductive stage, forty chickpea genotypes, including drought-resistant types, released varieties and advanced breeding lines, were obtained from Lead Centre, All India Coordinated Research Project (AICRP) and the Department of Plant Breeding Genetics, Jawaharlal Nehru Krishi Vishwa Vidyalaya, Jabalpur, Madhaya Pradesh, India (Supplementary Material Table S1). The field was prepared with 1 m wide bed flanked by 0.45 m furrows and fertilized with di-ammonium phosphate (DAP) containing nitrogen (18.0 kg/ha) and phosphorus (20.0 kg/ha). Seeds were treated with Bavistin (2.0 g per kg seed weight), Chlorpyriphos 20EC (10.0 mL per kg seed) and Rhizobium (5.0 g per kg seed). Seeds were sown at a depth of 2–3 cm manually, maintaining a row-to-row distance of 45 cm. For the uniform emergence of seedlings, 20 mm irrigation was applied immediately after sowing. Thinning was performed after two weeks of seed germination to maintain a plant-to-plant distance of 10 cm within rows. Subsequently, drought stress was imposed by withholding the water supply to the stressed set of plots before the onset of pod initiation up to the harvesting [29]. 

### 2.1. Physiological Traits

Relative water content (RWC) and canopy temperature depression (CTD) were estimated according to Gontia-Mishra et al. [30] and Purushothaman et al. [26], respectively. The leaf gas exchange parameters, viz., photosynthesis rate (Pn), stomatal conductance (g_s_), transpiration rate (Tr) and internal CO_2_ concentration (Ci), were recorded using a portable infra-red gas analyzer (IRGA) LiCor-6400 (LiCor Instruments, Lincol, NE, USA).

### 2.2. Biochemical Traits

Chlorophyll content was estimated according to Gontia-Mishra et al. [30], while protein content was determined using an extraction buffer, as mentioned in the Bradford assay [31]. To determine the oxidative stress of a cell, hydrogen peroxide (H_2_O_2_) content, lipid peroxidation content (malondialdehyde; MDA) and electrolyte leakage (EL) were measured as described by Velikova et al. [32], Naservafaeito et al. [33] and Sachdeva et al. [28], respectively. To estimate the osmolytes accumulation of a cell, free proline content of leaf using ninhydrin [30] and total soluble sugar content using an anthrone reagent methodology [34] were determined.

To determine the enhanced activity of antioxidant enzymes, crude enzyme was extracted using an enzyme extraction buffer. Superoxide dismutase (SOD) activity was determined according to Sharma et al. [35], and one unit of enzyme activity was defined as the amount of enzyme that decreased the absorbance by 50%. The estimation of peroxidase (POD) activity was performed following Rao et al. [36], and enzyme activity was calculated as per extinction coefficient of tetra-guaiacol ∈ = 26.6 mM^−1^ cm^−1^. Catalase (CAT) activity was estimated according to Aebi et al. [37], and enzyme activity was calculated as the amount of H_2_O_2_ decomposed per min. Ascorbate peroxidase (APX) activity was determined as described by Nakano et al. [38], and enzyme activity was calculated as per extinction coefficient of ascorbate ∈ = 2.8 mM^−1^cm^−1^.

### 2.3. Morpho-Phenological Traits, Yield and Yield Attributing Traits

Plant height was recorded from the ground level to the shoot tip. The date when half of the plants in a replication had at least one flower opened and the date when more than 75% of the pods of a plant turned brownish yellow from the days after sowing (DAS) were recorded as days to 50% flowering (DTF) and days to maturity (DTM), respectively. At the time of harvesting, all the seed-filled pods of a plant were counted as numbers of pods (NOP), and the weight of the plant including the pods was recorded as biological yield per plant (BYPP). The harvested seeds of a plant were weighed to obtain seed yield per plant (SYPP). Harvest index (%) was calculated as the ratio between seed yield per plant and biological yield per plant multiplied by 100. 

### 2.4. Statistical Analysis 

From each treatment, three plants were randomly selected to record the various drought-related morpho-phenological, physio-biochemical, and yield traits in two successive Rabi seasons (2020–2021 and 2021–2022). The data of both seasons were pooled for all 40 chickpea genotypes under both water conditions. The significance was established by analysis of variance (ANOVA) and Duncan Multiple Range Test (DMRT) at *p* < 0.05 using STAR V2.0.1 and SPSS V20 software, respectively. Principle component analysis (PCA) and PCA-based biplots were constructed to select reliable chickpea genotype (s) under drought-stressed conditions using XLSTAT software. Cluster analysis was also constructed, employing algometric hierarchical clustering for all chickpea genotypes under drought stress by applying STAR V2.0.1. 

## 3. Results 

### 3.1. Effect of Terminal Drought Stress on Physiological Traits

**Figure 1:**
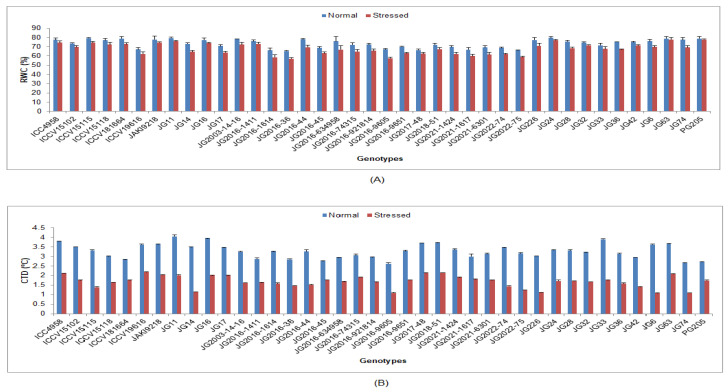
Effect of terminal drought stress on (**A**) RWC and (**B**) CTD of studied chickpea genotypes, where RWC and CTD indicate relative water content and canopy temperature depression, respectively.

**Figure 2:**
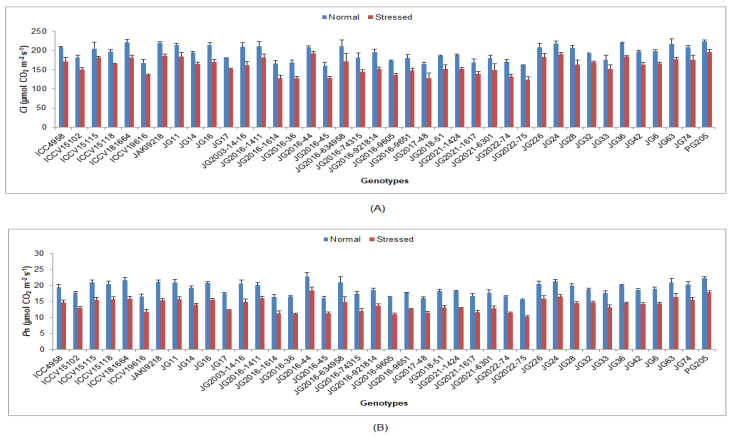
Effect of terminal drought stress on (**A**) *C*i (**B**) *P*n of studied chickpea genotypes, where *C*i and *P*n indicate internal CO_2_ concentration and photosynthesis rate, respectively.

**Figure 3:**
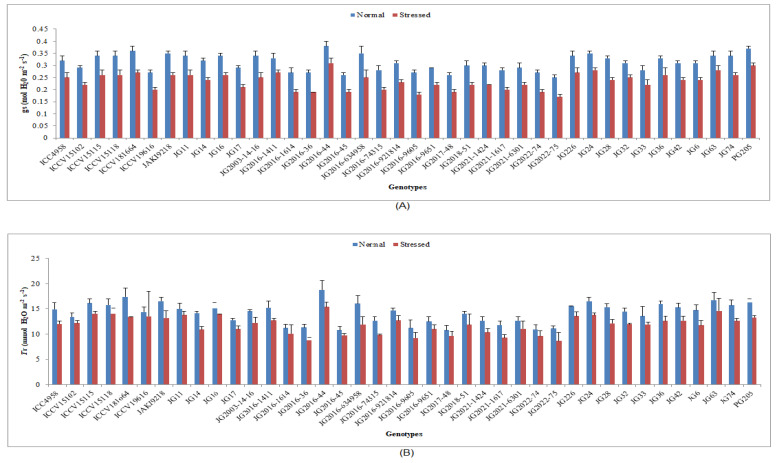
Effect of terminal drought stress on (**A**) gs and (**B**) *T*r of studied chickpea genotypes, where gs and *T*r indicate stomatal conductance and transpiration rate, respectively.

Under terminal drought-stressed condition, all studied physiological traits were significantly decreased in comparison to normal irrigated conditions in all chickpea genotypes (Supplementary Materials Tables S2 and S3). A higher RWC was maintained by genotype JG63 (77.66%), whereas lower RWC was noted in the genotype JG2016-36 (57.03%) (Figure 1). Higher CTD was obtained by genotype ICCV19616 (2.18 °C), whereas the lowest CTD was reported in genotype JG6 (1.08 °C). In terms of *C*i, the highest value was achieved in genotype PG205 (195.9 µmol CO_2_ m^−2^s^−1^), whereas the lowest *C*i was recorded in genotype JG2022-75 (123.78 µmol CO_2_ m^−2^s^−1^) (Figure 2). Figure 2 and Figure 3 show that genotype JG2016-44 exhibited the maximum *P*n (18.31 μmol CO_2_ m^−2^s^−1^) and gs (0.31 mol H_2_O m^−2^s^−1^), while the minimum *P*n (10.31 μmol CO_2_ m^−2^s^−1^) and gs (0.17 mol H_2_O m^−2^s^−1^) were found in genotype JG2022-75. Higher *T*r was maintained in genotype JG2016-44 (15.4 mmol H_2_O m^−2^s^−1^),and the lowest was seen in genotype JG2022-75 (8.62 mmol H_2_O m^−2^s^−1^).

### 3.2. Effect of Terminal Drought Stress on Biochemical Traits

**Figure 4:**
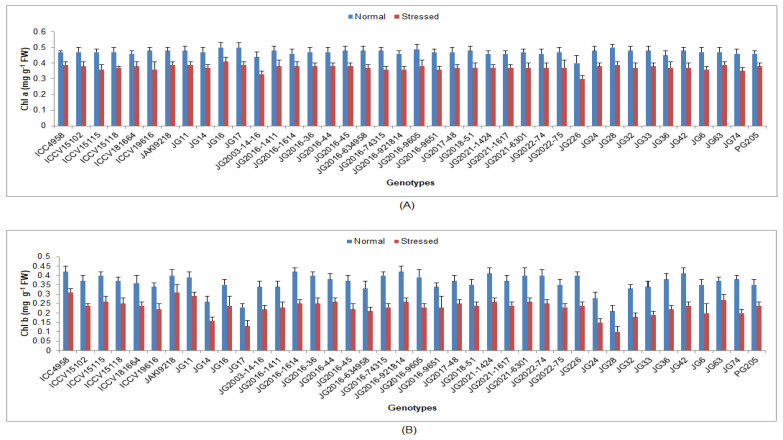
Effect of terminal drought stress on (**A**) Chl a and **(B**) Chl b content of studied chickpea genotypes, where Chl a and Chl b indicate chlorophyll a and chlorophyll b, respectively.

**Figure 5:**
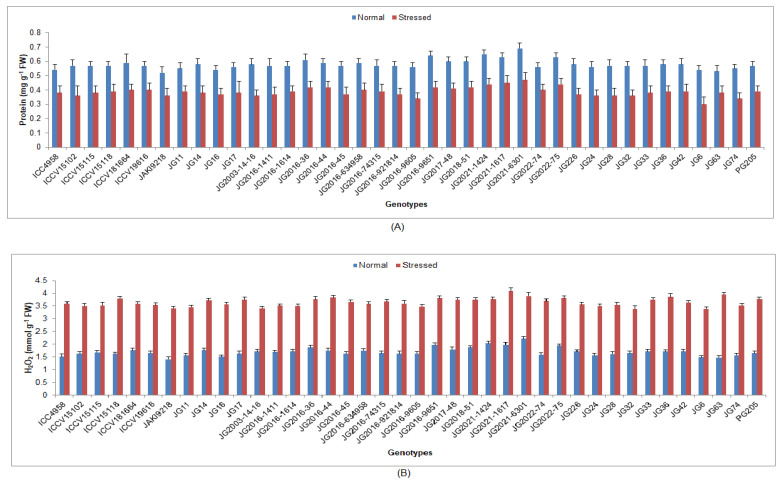
Effect of terminal drought stress on (**A**) protein content and (**B**) H_2_O_2_ content of studied chickpea genotypes, where H_2_O_2_ indicates hydrogen peroxide.

**Figure 6:**
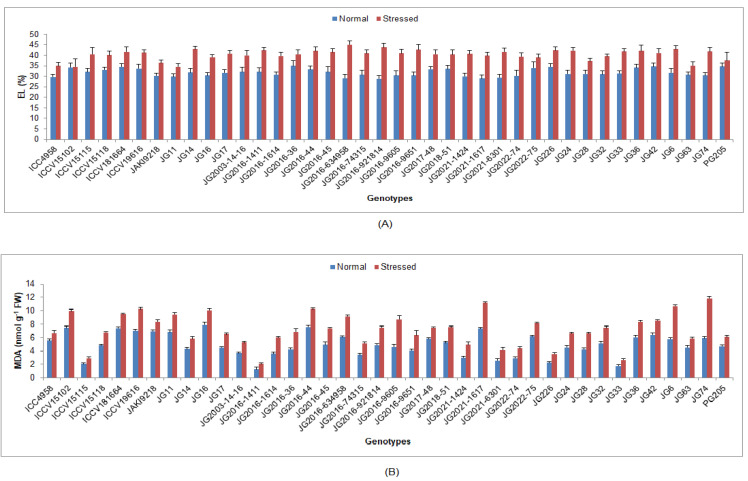
Effect of terminal drought stress on (**A**) EL (%) and (**B**) MDA content of studied chickpea genotypes, where EL and MDA indicate electrolyte leakage and malondialdehyde, respectively.

**Figure 7:**
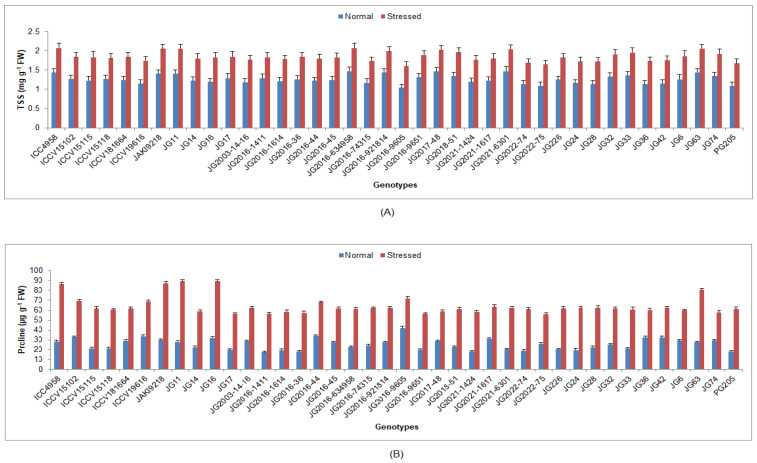
Effect of terminal drought stress on (**A**) TSS content and (**B**) proline content of studied chickpea genotypes, where TSS indicates total soluble sugar.

Under terminal drought-stressed conditions, chlorophyll a, b and protein content were significantly reduced, whilst H_2_O_2_ content, EL, lipid peroxidation (MDA) and antioxidant enzyme activities were significantly enhanced as compared to normal irrigated condition in all investigated chickpea genotypes (Supplementary Tables S4 and S5). Higher Chl ‘a’ was maintained by genotype JG16 (0.41 mg/g FW) (Figure 4), while higher Chl ‘b’ by genotype ICC4958 (0.31 mg/g FW). Higher protein content was upheld by genotype JG2021-6301 (0.47 mg/g FW), whilst the minimum was documented in genotype JG74 (0.34 mg/g FW) (Figure 5). Minimum H_2_O_2_ content was recorded in genotype JG6 (3.39 mmol/g FW), while maximum enrichment in H_2_O_2_ was found in genotype JG2021-6301 (42.93%). Higher EL was observed in genotype JG2016-634958 (45.08%), whilst the minimum was found in genotype JG11 (34.49%) (Figure 6). Minimum MDA content was noticed in genotype JG2016-1411 (2.05 nmol/g), whereas the maximum was documented in genotype ICC4958 (16.29%). Higher TSS content was maintained by genotype ICC4958 (2.07 mg/g FW), whereas the lowest was recorded in genotype JG2016-9605 (1.60 mg/g FW) (Figure 7). Higher proline content was detected in genotype JG11 (89.18 µg/g FW), while the lowest was noticed in genotype JG2022-75 (55.83 µg/g FW). 

**Figure 8:**
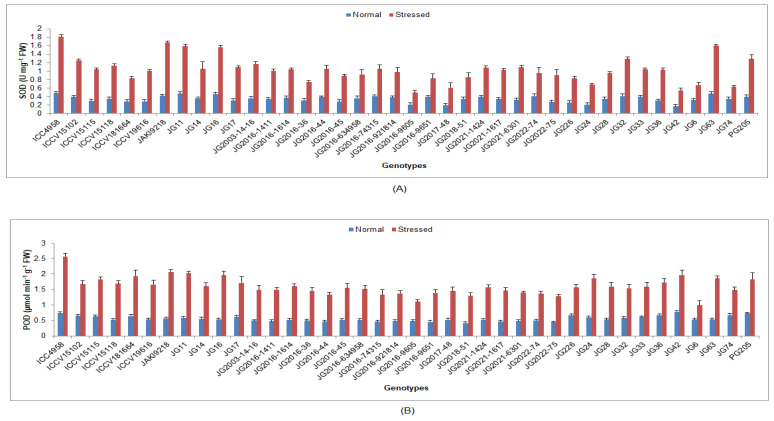
Effect of terminal drought stress on (**A**) SOD and (**B**) POD activity of studied chickpea genotypes, where SOD and POD indicate superoxide dismutase and peroxidise.

**Figure 9:**
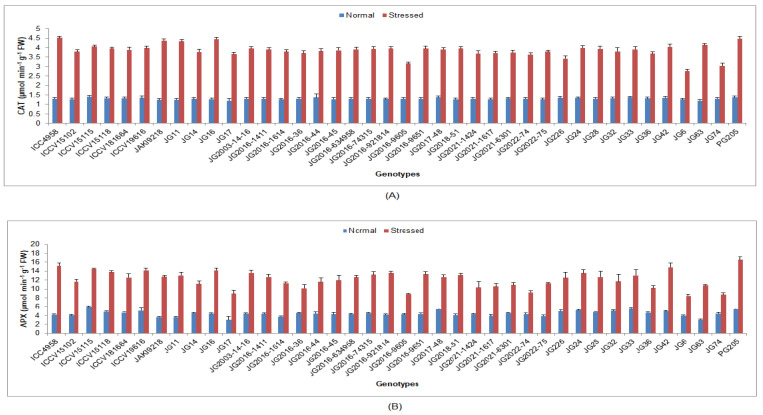
Effect of terminal drought stress on (**A**) CAT and (**B**) APX activity of studied chickpea genotypes, where CAT and APX indicate catalase and ascorbate peroxidise, respectively.

Higher SOD was maintained by genotype ICC4958 (1.82 U/mg FW), while the minimum was recorded for the genotype JG206-9605 (0.49 U/mg FW) (Figure 8). Higher POD was sustained in genotype ICC4958 (2.57 µmol/min/g FW), whilst the minimum was evidenced in genotype JG6 (0.99 µmol/min/g FW). Higher CAT was maintained by genotype ICC4958 (4.52 µmol/min/g FW), whereas the minimum was perceived in genotype (JG6 2.77 µmol/min/g FW) (Figure 9). Higher APX was exhibited by the genotypePG205 (16.54 µmol/min/g FW), whilst the lowest was found in genotype JG6 (8.43 µmol/min/g FW).

### 3.3. Effect of Terminal Drought Stress on Yield and Its Attributing Traits

**Figure 10:**
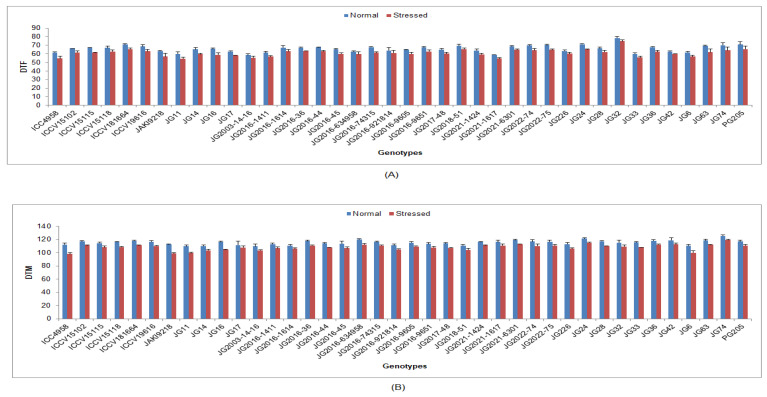
Effect of terminal drought stress on (**A**) DTF and (**B**) DTM of studied chickpea genotypes, where DTF and DTM indicate days to 50% flowering and days to maturity, respectively.

**Figure 11:**
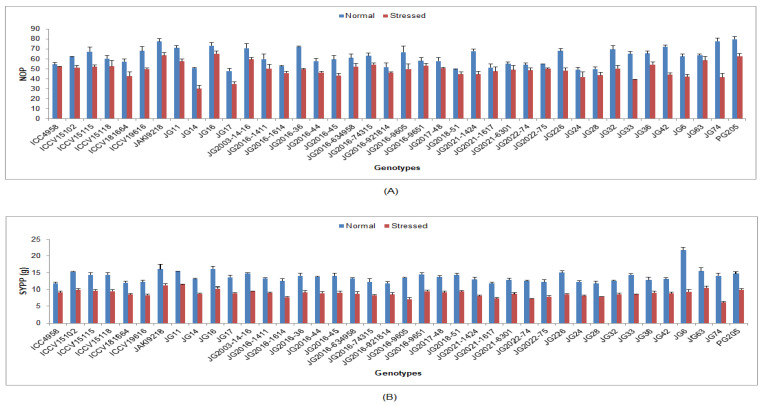
Effect of terminal drought stress on (**A**) NOP and (**B**) SYPP of studied chickpea genotypes, where NOP and SYPP indicate number of pods and seed yield per plant, respectively.

**Figure 12:**
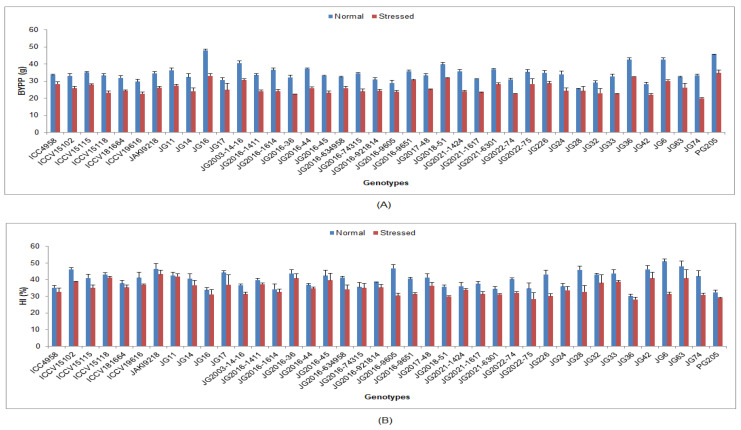
Effect of terminal drought stress on (**A**) BYPP and (**B**) HI (%) of studied chickpea genotypes, where BYPP and HI indicate biological yield per plant and harvest index, respectively.

Under the drought-stressed condition, yield and its accrediting characters were significantly reduced in all the studied chickpea genotypes compared to the normal irrigated condition (Supplementary Tables S6 and S7). In terms of genotypic response, the lowest DTF was documented in genotype JG11 (54.3 DAS), while the maximum was in genotype JG32 (74.91DAS) (Figure 10). Lower DTM was documented in genotype ICC4958 (98.13 DAS), whilst maximum DTM was investigated in genotype JG74 (119.42 DAS). Higher NOP was maintained by genotype JG16 (65.25), whereas the minimum was observed in genotype JG14 (30.25) (Figure 11). Higher SYPP was upheld by genotype JG11 (11.42 g), the while minimum was observed in genotype JG74 (6.14 g). Higher BYPP was sustained in genotype PG205 (34.77 g), and the minimum was shown in genotype JG74 (19.33 g) (Figure 12). Higher HI was exhibited by the genotype JAKI9218 (43.29%), whereas the minimum was found in genotype JG36 (28.18%).

### 3.4. Principle Component Biplot Analysis

**Figure 13:**
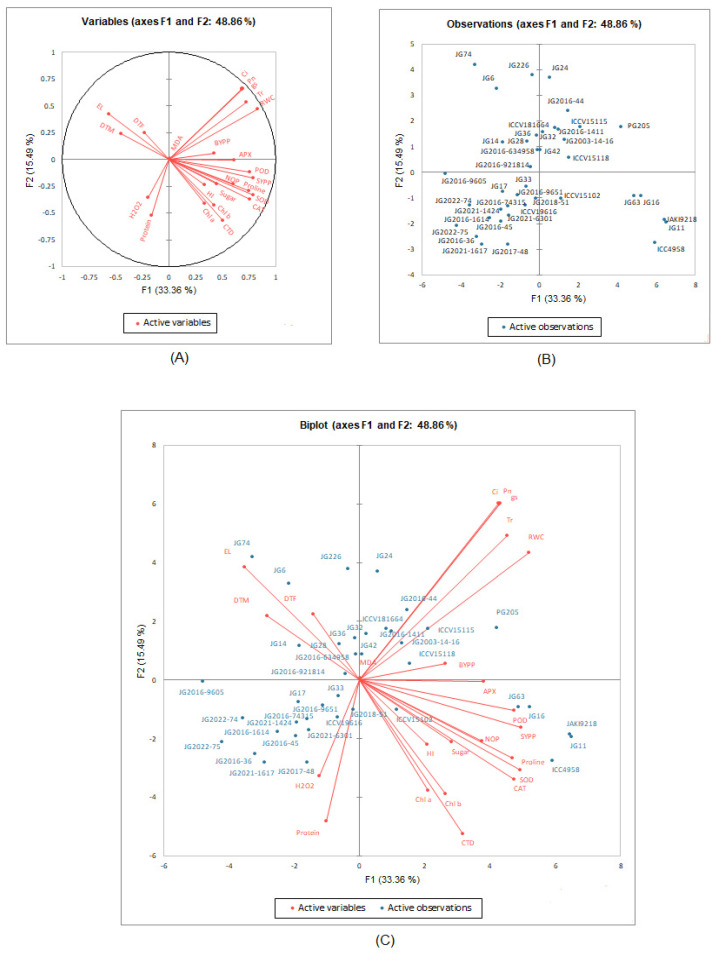
PCA biplots depicting (**A**) relationships between the traits measured, (**B**) performance of chickpea genotypes, and (**C**) combined (**A** + **B**) under terminal drought stressed condition. In the active variables, RWC, CTD, Ci, Pn, gs, Tr, Chla, Chlb, EL, MDA, H_2_O_2_, SOD, POD, APX, DTF, DTM, NOP, SY, BY, and HI indicate the relative water content, canopy temperature depression, internal CO_2_ concentration, photosynthesis rate, stomatal conductance, transpiration rate, chlorophyll a, chlorophyll b, electrolyte leakage, malondialdehyde, hydrogen peroxide, superoxide dismutase, peroxidase, ascorbate peroxidase, days to 50% flowering, days to maturity, number of pods, seed yield, biological yield and harvest index, respectively.

For a more reliable identification of genotypes with a maximum value for one or more traits, genotype by trait biplots were constructed for PC-I and PC-II for all genotypes and all traits under all treatments (Figure 13, Supplementary Table S8). Biplot analysis clearly distinguished the drought-associated traits into positively correlated traits (<90°), independent traits (=90°), and negatively correlated traits (>90°). The RWC, CTD, *P*n, *g*s, and *C*i were identified as positively correlated traits among the studied physiological traits; chl a, chl b, TSS and proline contents, including antioxidant enzymes activities, viz., SOD, POD, CAT, and APX, were proved to be positively correlated traits among the studied biochemical traits. Similarly, SYPP, NOP, and BYPP were also considered positively correlated traits among the studied yield and its attributes. These cumulative positively correlated physio-biochemical traits, yield, and its attributing traits contributed more towards the drought tolerance of chickpea genotypes, and so can be treated as markers for terminal drought tolerance in chickpea.

**Figure 14:**
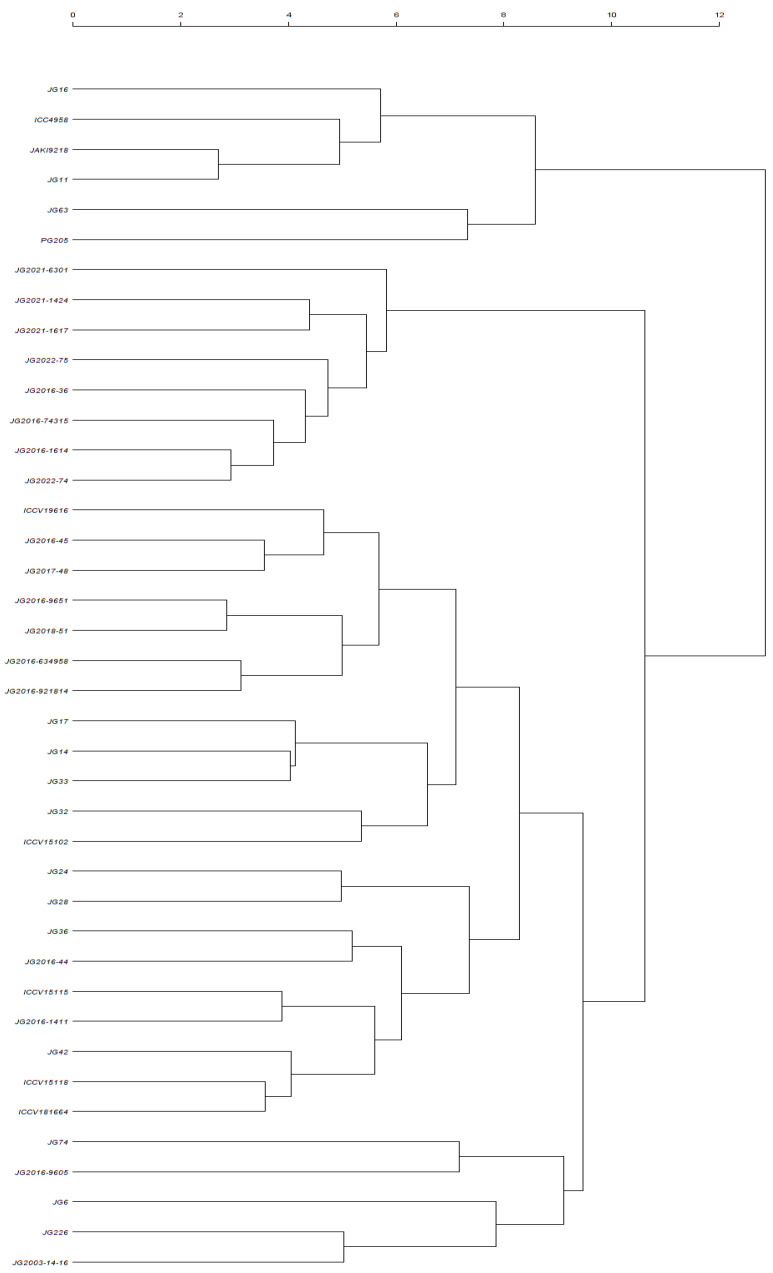
Agglomerative clustering of studied chickpea genotypes under terminal drought-stressed condition.

In biplots, the genotypic performance can be estimated by the distance of the genotype from the origin of the biplot. The distant genotypes could have the greatest values for one or more traits. The PCA biplot distinguishes the ICC4958, JG11, JAKI9218, JG16, and JG63 genotypes as distant genotypes with strong positive correlation with CAT, SOD, proline, TSS, POD, and APX selection indices. These genotypes could have the greatest values for these selection indices. The bilpot also distinguishes JG6 and JG74 genotypes as distant genotypes with strong negative correlation with these selection indices. These genotypes could have minimum values for these selection indices. The rest of the genotypes could have medium values for these selection indices. Further, cluster analysis was performed using morpho-physiological and biochemical data under the stress condition. The agglomerative clustering categorized forty genotypes into two major clusters (Figure 14). Major cluster I consisted of six genotypes, viz., JG16, ICC4958, JAKI9218, JG11, JG63, and PG205. Major cluster II consisted of two subclusters. Sub-cluster I also contained six genotypes, viz., JG74, JG2016-9605, JG6, JG226, and JG-2003-14-16Sub-cluster II contained the rest of the genotypes.

## 4. Discussion

Abiotic stresses are almost interlinked, causing morpho-physiological, biochemical, and molecular alteration that negatively affects crop growth and development, crop efficiency, and ultimately yield [4,20,21,22]. The prevalence of inconsistent rainfall and extreme temperature (drought and heat) is proposed to increase soon owing to climate change [39]. Low moisture and heat affect chickpea growth and may be observed in early morphological stages that ultimately affect seed yield indirectly. The present findings revealed that under normal sown conditions, there was a substantial increase in plant growth and development compared to under drought-stressed conditions. 

Plant water status is the primary factor that affects crop yield and quality. The present investigation reveals that drought stress caused a significantly reduced RWC content in the leaves of genotypes. RWC decreased in lesser magnitude in drought-tolerant genotypes; this may be because of their more extended root systems, which could complement water lost by transpiration. Under drought-stressed conditions, the ability of a plant to maintain the turgor pressure and related physiological processes has great significance, and it is related to drought resistance in terms of osmoregulatory activities. Drought stress leads to the dehydration of plants and a decline in RLWC, which can result in stomatal closure [40,41,42,43]. CTD was also decreased under the stressed conditions compared to normal conditions. The drought-tolerant chickpea genotypes demonstrated higher CTD under drought-stressed conditions than other genotypes, showing their extraordinary ability to maintain a canopy cooler than the rest. CTD has already been utilized as a selection indicator for tolerance to drought and high-temperature stress in early-generation selections [44,45]. A positive correlation of CTD with yield was also observed in chickpea under heat-stressed and drought-stressed conditions [26]. Various other studies have also described a comparable pattern of decreases in CTD under heat- and water-stressed conditions in chickpea genotypes [26,39].

Under terminal drought-stressed condition, the gas-exchange parameters were also decreased in all studied chickpea genotypes. The most negligible reduction was evidenced in tolerant genotypes compared to other genotypes. The decrease in internal CO_2_ concentration and leaf photosynthetic rate under drought-stressed conditions appears to be mediated by stomatal closure, as demonstrated by the reduced stomatal conductance and transpiration rate [46,47]. In this investigation, pigment and protein content were also reduced under stress conditions, and less reduction was documented in tolerant genotypes compared to other genotypes. Chl ‘a’, Chl ‘b’ and total chlorophyll content in chickpea leaves was shown to be degraded with increasing days of irrigation intervals compared with unstressed plants. The water deficit condition decreased chickpea growth, chlorophyll content and photosynthetic efficiency when plants were exposed to irrigation levels of 100, 60, 40 and 20% of the field capacity [48]. Protein molecules play a crucial role in the proper functioning of the cell. In this study, protein content decreased in all genotypes under drought stress, and the most negligible reduction was detected in tolerant genotypes compared to other genotypes [49]. Reduced photosynthesis under drought stress reduces or even stops protein synthesis. Abiotic stresses caused a reduction in protein production, possibly due to various factors involved [50]. 

Water stress enhances the production of ROS such as alkoxy radicals, singlet oxygen, O_2_^•−^, OH^•^, H_2_O_2_, etc. Increased H_2_O_2_ content induces oxidative stress with several adverse effects, including electrolyte leakage, associated membrane damage, and lipid peroxidation. In this research, tolerant genotypes showed a lesser increase in H_2_O_2_, EL and MDA contents than other genotypes. Under drought stress, similar findings of increased leaf H_2_O_2_ [44], EL and MDA content [39,41] were also reported in chickpea. Under terminal drought-stressed conditions, the chickpea genotypes accumulated osmolytes. Drought-tolerant genotypes accumulated higher osmolyte levels, suggesting that osmolytes might be proved an appropriate indicator for evaluating drought tolerance in chickpea. In the shoots of the chickpea plants, proline content was significantly increased under moderate and severe drought-stressed conditions compared with untreated plants [50]. Although water stress induced a significant increase in leaf proline content of the sensitive cultivar (Azad), leaf proline content in the tolerant cultivar (Arman) strongly increased [48]. Owing to unpredicted changes in climate, pulses become more sensitive to oxidative damage by the overproduction of ROS such as H_2_O_2_, hydroxyl, and superoxide radicals. Specialized enzymatic antioxidants, i.e., POD, SOD, APX and CAT, are activated and act as the first line of defense for detoxification of the effects of ROS [51]. In the present study, increased activity of SOD, POD, CAT, and APX was investigated in all genotypes under drought-stressed conditions over the normal condition. A higher activity level was evidenced in tolerant genotypes compared to other genotypes. Several earlier researchers also reported a similar increased level of antioxidant enzyme activities under water stress conditions in chickpea. SOD, POX and catalase activities were significantly enhanced in moderate (50% FC) and severe (25% FC) conditions under drought stress [47]. CAT, SOD, POX, APX and GR activities were markedly increased in chickpea shoots under moderate and severe drought-stressed conditions [46]. CAT, SOD, POX, APX and GR activities were markedly enhanced in chickpea plants under drought stress [4,48,50] circumstances as well.

Under the normal irrigated condition, the maximum grain yield per plant was documented by genotype JG6, tracked by the genotypes JG16, JAKI9218, and JG11. The maximum yield per plant was yielded by genotype JG11, tailed by genotypes JAKI9218, JG16, and ICC4958 under terminal drought stress. In this investigation, the tolerance of genotypes JG11, JAKI9218, JG16, and ICC4958 against drought stress was perhaps due to the higher number of pods per plant, the better accumulation of osmolytes, i.e., sugar and proline, and the greater activities of antioxidant enzymes, viz., SOD, POD, CAT, and APX. Similarly reduced yield attributes including the numbers of pods and numbers of seeds per plant, and hundred-seed weight under moderate and severe drought-stressed conditions have also been observed in chickpea, allowing us to conclude that the synthesis of enzymatic and non-enzymatic antioxidants and proline content in stressed plants helped in the protection of plants under drought-stressed conditions [50]. A significant difference was investigated among the genotypes based on different biochemical, morphological, and physiological parameters. The chickpea genotypes, viz., GGP-1260, PGP-1426, and PB-1, were considered drought-tolerant genotypes based on their higher plant biomass production, pod yield, harvest index, and having the highest activities of POD, CAT, and SOD. Under drought stress, the drought-tolerant genotypes retained higher plant yield with lower reductions in CI, RWC, MSI, numbers of secondary branches, and biomass [28]. An integrated approach involving physio-biochemical traits and multi-environmental yield trials was performed for screening and selecting drought-tolerant chickpea genotypes and allowed us to conclude that higher RWC, CMS, glycine betaine, and proline content conferred a more significant capability for drought stress tolerance in chickpea [16]. In another investigation, the reduction in growth and yield of the tolerant cultivar was less compared to the susceptible cultivar DUSHT, probably due to the accumulation of higher antioxidant enzyme activities, suggesting the protective role of enhanced antioxidant enzyme activities of plants under water-stressed conditions [51].

PCA biplot is the most effective multivariate analysis for evaluating the genotypic performance and traits interaction. It is being extensively utilized to dissect the traits correlation in different crop plants by several researchers [16]. PCA biplots provided a new understanding of drought-tolerance mechanisms and plant responses under drought-stress conditions [28]. Under the stressed condition, biplot analysis based on principal component and correlation analysis established a strong positive association of SYPP with POD, NOP, proline, SOD, CAT, APX, and sugar content, signifying their greater utilization in selecting high-yielding drought-tolerant genotypes. Genotypes ICC4958, JAKI9218, JG11, JG16, and JG63 performed better under the stressed condition, with a smaller reduction in NOP and BYPP, including a higher accumulation of osmolytes (proline and sugar) and enhanced antioxidant enzyme (POD, SOD, APX, and CAT) activity. Further, the agglomerative clustering also supported the result obtained from biplot analysis and grouped tolerant and susceptible genotypes in separate clusters. Major cluster I contained tolerant genotypes, while sub-cluster I consisted of susceptible genotypes. Our findings follow the results of Sachdeva et al. [28], who also observed a strong positive association with RWC, chlorophyll index (CI), membrane stability index (MSI), numbers of secondary branches (SB) and yield traits and negative associations with drought-susceptibility index (DSI), 100-SW and days to maturity under drought-stressed conditions through principal component analysis based on biplot and correlation analysis. Genotypes ICC4958, Pusa1103, BGD72, CSG8962, ICCV97309, ICCV10, ICCV03311, ICCV05308, ICCV3403, and ICCV10313 were identified as the most drought-tolerant genotypes, with higher values of lower DSI and DTM and high RWC and MSI values under drought-stressed conditions at both vegetative and reproductive stages based on PCA-biplot analysis. Similarly, Shah et al. [16] also utilized biplot analysis to select superior chickpea genotypes under drought stress and concluded that genotypes D0091–10, D0085–10, K010–10, K005–10, 08AG016, D0078–10, 08AG004, 09AG002, D0080–10, K002–10 and D0099–10 proved superior in yield as well as physio-biochemical performances under drought-stressed multiple environmental conditions. Furthermore, genotype by-trait (GT) biplots were constructed for a more reliable identification of genotype with maximum value for multiple traits in chickpea for all genotype under stress conditions [4].

## 5. Conclusions

The identification of new genetic resources that are tolerant to drought-stressed conditions is crucial. However, simultaneously, attention has been given to identifying suitable physiological and biochemical markers that can be employed to distinguish the tolerant and susceptible genotypes. The PCA biplots revealed that POD, NOP, proline, SOD, APX, CAT and sugar content showing strong positive association with SYPP could be used as selection indices to distinguish between tolerant and sensitive genotypes. ICC4958, JAKI9218, JG11, JG16, JG63, and PG205 performed better in the terminal drought-stressed condition with higher accumulation of proline and sugar, enhanced activity of POD, SOD, APX, and CAT enzyme activities and smaller reduction in NOP. Due to the unavailability of quantitative real-time polymerase chain reaction (qRT PCR), expression analysis of drought-associated genes could not be performed. So, further analysis of gene expression and the nutritional profiling of drought-tolerant chickpea genotypes may be performed to further explore the genetic traits of the selected drought-tolerant genotypes.

## Data Availability

Not applicable.

## Supplementary Materials

The following supporting information can be downloaded at: https://www.mdpi.com/article/10.3390/life13061405/s1. Table S1. Details of the 40 chickpea genotypes used for present study. Table S2. Pooled physiological responses of various chickpea genotypes under normal irrigated condition. Table S3. Physiological responses of various chickpea genotypes under terminal drought stressed condition. Table S4. Pooled biochemical responses of various chickpea genotypes under normal irrigated condition. Table S5. Biochemical responses of various chickpea genotypes under terminal drought stressed condition. Table S6. Yield and yield attributing trait responses of various chickpea genotypes under normal irrigated condition. Table S7. Yield and yield attributing trait responses of various chickpea genotypes under terminal drought stressed condition. Table S8. PC Scores of studied chickpea genotypes under terminal drought stressed condition.
