# Supplementary material for: Screening and Selection of Drought-Tolerant High-Yielding Chickpea Genotypes Based on Physio-Biochemical Selection Indices and Yield Trials
Source: Life (Basel). 2023 Jun 17;13(6):1405. doi: 10.3390/life13061405 (PMC10302310; doi:10.3390/life13061405)
Supplement: Supplementary file 1 [file life-13-01405-s001.zip › life-2389234-supplementary.pdf]

Supplementary Material Table S1. Details of the 40 chickpea genotypes used for present study.

S. No.	Genotype Name	Status	Pedigree	Source
1	ICC4958	National check for drought tolerance	JGC 4958	JNKVV, Jabalpur
2	JAKI9218	Released variety	(ICCC 37 x GW5/7) x ICCV 107	JNKVV, Jabalpur
3	JG11	Released variety	(Phule G-5 x Narsinghpur bold) x ICCC 37	JNKVV, Jabalpur
4	JG16	Released variety	ICCC 44 × ICCV 10	JNKVV, Jabalpur
5	JG63	Released variety	Single Plant selection from JG 62	JNKVV, Jabalpur
6	JG74	Released variety	A composite from genetic stock	JNKVV, Jabalpur
7	JG6	Released variety	(ICCV10XK850) x (H208XRS11)	JNKVV, Jabalpur
8	JG14	Released variety	(GW5/7XP326) XICCL83149	JNKVV, Jabalpur
9	JG17	Released variety	BDNG 9-3 x Narshingpur Bold	JNKVV, Jabalpur
10	JG24	Released variety	(JG 74 x ICC 4958)-21	JNKVV, Jabalpur
11	JG28	Released variety	[(JM – 1 X IPC 9239) X JG 7] – 14-11	JNKVV, Jabalpur
12	JG32	Released variety	[(JM – 1 x IPC 4958) x JG 315] – 2	JNKVV, Jabalpur
13	JG33	Released variety	[(JM – 1 x IPC 9239) x JG 322] – 30-3	JNKVV, Jabalpur
14	JG36	Released variety	JG 12 x JG 16	JNKVV, Jabalpur
15	JG42	Released variety	[(JM 1x IPC 9239) JG7] 14-11-2011-42	JNKVV, Jabalpur
16	JG226	Released variety	JG 74 x JG315	JNKVV, Jabalpur
17	JG205	Released variety	JG 315 x ICC 96029	JNKVV, Jabalpur
18	ICCV15102	Released variety	ICCV03112 x ICCV10	ICRISAT, Patancheru
19	ICCV15115	Released variety	ICCV10 x ICCV 96970	ICRISAT, Patancheru
20	ICCV15118	Released variety	ICCV 05530 x ICCV 88510	ICRISAT, Patancheru
21	ICCV19616	Released variety	JAKI 9218/ICCV 05103	ICRISAT, Patancheru
22	ICCV181664	Released variety	ICC 4958 TM/JG 130	ICRISAT, Patancheru
23	JG2003-14-16	Advanced breeding line	[(JM1 x ICC4929) x ICC4958]-2-14-16	JNKVV, Jabalpur
24	JG2016-44	Advanced breeding line	(ICC 96029 x ICC11551) 44	JNKVV, Jabalpur

25	JG2016-45	Advanced breeding line	(JG 74 x ICC11551) 45	JNKVV, Jabalpur
26	JG2016-1411	Advanced breeding line	JG 14 x JG 11	JNKVV, Jabalpur
27	JG2016-1614	Advanced breeding line	JG 16 x JG 14	JNKVV, Jabalpur
28	JG2016-9605	Advanced breeding line	JG 74 x ICC 96029	JNKVV, Jabalpur
29	JG2016-9651	Advanced breeding line	JG 130 x ICC 96029	JNKVV, Jabalpur
30	JG2016-74315	Advanced breeding line	[{(JG 74 x WR 315) x JG 74} -2010 -1- 3- 5- 11-15-10-2]	JNKVV, Jabalpur
31	JG2016-634958	Advanced breeding line	JG 63 x ICC 4958	JNKVV, Jabalpur
32	JG2016-921814	Advanced breeding line	JAKI 9218 x JG 14	JNKVV, Jabalpur
33	JG2017-48	Advanced breeding line	(JG 315 x ICC 96029)48	JNKVV, Jabalpur
34	JG2018-51	Advanced breeding line	JG63 x ICC1205	JNKVV, Jabalpur
35	JG2022-74	Advanced breeding line	JG12XJG74	JNKVV, Jabalpur
36	JG2016-36	Advanced breeding line	JG12XJG16-1	JNKVV, Jabalpur
37	JG2022-75	Advanced breeding line	JG12XICC4958	JNKVV, Jabalpur
38	JG2021-6301	Advanced breeding line	JG12 X ICCV06301	JNKVV, Jabalpur
39	JG2021-1424	Advanced breeding line	JG14XJG24	JNKVV, Jabalpur
40	JG2021-1617	Advanced breeding line	JG16 X JG17	JNKVV, Jabalpur

Supplementary Material Table S2. Pooled physiological responses of various chickpea genotypes under normal irrigated condition.

Genotypes	RWC	CTD	SLA	Ci	Pn	gs	Tr
ICC4958	77.35±2.23^{mnop}	3.79±0.01^{s}	180.5±1.39^{de}	208.02±3.33^{jklmn}	19.34±0.96^{hijklmn}	0.32±0.02^{hijk}	14.93±1.33^{fghijkl}
ICCV15102	73.49±0.83^{ghijkl}	3.5±0.01^{o}	215.83±2.9^{i}	181.86±5.87^{defg}	17.71±0.49^{defg}	0.29±0.01^{defg}	13.42±0.73^{cdef}
ICCV15115	79.19±0.61^{op}	3.32±0.03^{lmn}	253.73±2.83^{q}	204.73±17.66^{ijklm}	20.87±0.87^{no}	0.34±0.02^{lmn}	16.14±0.84^{ijklm}
ICCV15118	76.96±1.92^{mnop}	3±0.02^{fg}	228.95±2.37^{jk}	197.61±4.15^{hijkl}	20.49±0.83^{lmno}	0.34±0.02^{jklm}	15.79±1.24^{hijklm}
ICCV181664	78.76±2.11^{op}	2.83±0.02^{d}	228.95±1.74^{jk}	220.88±7.72^{no}	21.59±0.9^{opq}	0.36±0.02^{mno}	17.39±1.74^{mn}
ICCV19616	67±1.86^{abc}	3.62±0.02^{p}	180.34±1.64^{de}	167.9±8.45^{abcd}	16.53±0.74^{abcd}	0.27±0.01^{abcd}	14.38±1.02^{defghij}
JAKI9218	77.64±4.03^{mnop}	3.63±0.02^{pq}	210.71±4.86^{h}	220.05±3.98^{no}	21.03±0.67^{nop}	0.35±0.01^{lmn}	16.5±0.81^{klm}
JG11	79.32±1.26^{op}	4.04±0.09^{u}	196.98±3.26^{fg}	214.83±3.97^{mno}	20.96±0.82^{nop}	0.34±0.02^{lmn}	15.01±1.15^{fghijkl}
JG14	72.95±1.26^{fghijk}	3.48±0.01^{o}	239.66±4.01^{mn}	194.29±4.28^{ghij}	19.26±0.54^{hijklm}	0.32±0.01^{hijk}	14.17±0.29^{defghi}
JG16	77.18±2.34^{mnop}	3.93±0.01^{t}	170.11±1.62^{c}	214.36±6.03^{mno}	20.73±0.39^{mno}	0.34±0.01^{lmn}	15.06±1.14^{fghijkl}
JG17	70.84±1.06^{defg}	3.45±0.02^{o}	242.96±6.28^{n}	180.64±0.95^{cdefg}	17.58±0.26^{defg}	0.29±0.01^{cdef}	12.78±0.38^{bcde}
JG2003-14-16	78.51±0.12^{nop}	3.24±0.04^{jk}	255.43±4.87^{q}	209.7±10.63^{klmn}	20.61±1.06^{lmno}	0.34±0.02^{klm}	14.53±0.35^{efghijk}
JG2016-1411	75.91±1.6^{klmnop}	2.87±0.06^{de}	147.32±2.12^{b}	211.65±11.27^{lmno}	20.07±0.81^{jklmn}	0.33±0.02^{ijkl}	15.21±1.37^{fghijkl}
JG2016-1614	66.18±2.12^{ab}	3.25±0.02^{jkl}	176.19±1.06^{d}	166.86±6.78^{abc}	16.35±0.82^{abcd}	0.27±0.02^{abcd}	11.2±0.72^{ab}
JG2016-36	65.2±0.58^{a}	2.84±0.03^{d}	210.48±4.35^{h}	169.45±5.66^{abcd}	16.44±0.34^{abcd}	0.27±0.01^{abcd}	11.33±0.63^{ab}
JG2016-44	78.09±0.75^{mnop}	3.26±0.08^{jklm}	193.38±1.52^{f}	209.35±2.49^{klmn}	22.79±1.23^{q}	0.38±0.02^{o}	18.76±1.86^{n}
JG2016-45	68.6±1.63^{abcd}	2.75±0.03^{c}	194.42±3.16^{f}	160.83±9.42^{a}	16.06±0.5a^{bc}	0.26±0.01^{abc}	10.79±0.74^{a}
JG2016-634958	75.96±5.05^{klmnop}	2.93±0.03^{ef}	198.35±1.36^{fg}	211.65±15.59^{lmno}	20.88±1.84^{no}	0.35±0.03^{lmn}	16.06±1.57^{hijklm}
JG2016-74315	71.97±2.25^{defghi}	3.05±0.07^{g}	233.09±3.29^{kl}	182.52±10.93^{defg}	17.32±0.86^{bcdef}	0.28±0.02^{bcdef}	12.65±0.78^{abcde}
JG2016-921814	72.35±1.44^{efghij}	2.95±0.02^{f}	234.37±3.5^{lm}	196.6±6.86^{hijk}	18.63±0.53^{fghi}	0.31±0.01^{fghi}	14.72±0.48^{fghijk}
JG2016-9605	67.17±0.82^{abc}	2.61±0.06^{a}	226.9±2.97^{j}	172.89±3^{abcde}	16.41±0.26^{abcd}	0.27±0.01^{abcd}	11.18±1.6^{ab}
JG2016-9651	69.95±0.85^{cdefg}	3.28±0.04^{klm}	200.68±2.37^{g}	180.03±9.48^{cdefg}	17.69±0.03^{defg}	0.29±0^{defg}	12.5±0.9^{abcd}
JG2017-48	66.45±1.29^{abc}	3.7±0.01^{qr}	217.68±1.46^{i}	165.19±4.37^{ab}	15.99±0.49^{ab}	0.26±0.01^{ab}	10.78±0.92^{a}
JG2018-51	72.14±1.65^{defghi}	3.71±0.01^{r}	221.14±2.28^{i}	185.58±1.92^{efgh}	18.14±0.72^{efgh}	0.3±0.02^{efgh}	14.05±0.48^{defgh}
JG2021-1424	69.81±1.52^{cdef}	3.36±0.04^{n}	235.42±3.85^{lm}	188.88±1.44^{fgh}	18.18±0.29^{fgh}	0.3±0.01^{efgh}	12.67±0.8^{abcde}
JG2021-1617	66.92±1.9^{abc}	2.99±0.13^{fg}	216.37±2.37^{i}	169.31±8.91^{abcd}	16.73±0.74^{abcde}	0.28±0.01^{abcde}	11.81±0.72^{abc}
JG2021-6301	69.23±2.13^{bcde}	3.13±0.04^{h}	167.76±4.42^{c}	180.45±7.27^{cdefg}	17.77±0.89^{defg}	0.29±0.02^{defg}	12.65±0.81^{abcde}
JG2022-74	68.7±0.62^{abcd}	3.45±0.02^{o}	182.99±2.39^{e}	170.45±6.91^{abcd}	16.48±0.32^{abcd}	0.27±0.01^{abcd}	10.93±0.9^{ab}
JG2022-75	66.1±0.59^{ab}	3.15±0.04^{hi}	248.26±3.45^{op}	162.15±0.44^{ab}	15.5±0.3^{a}	0.25±0.01^{a}	11.11±0.47^{ab}

JG226	77.15 ± 2.94^{mnop}	3.02 ± 0.01^{g}	175.25 ± 1.51^{d}	209.06 ± 10.4^{klmn}	20.41 ± 0.96^{lmno}	0.34 ± 0.02^{jklm}	15.53 ± 0.1^{ghijklm}
JG24	79.51 ± 1.66^{p}	3.33 ± 0.01^{mn}	239.33 ± 3.74^{mn}	218.86 ± 6.66^{mno}	21.2 ± 0.7^{nop}	0.35 ± 0.01^{lmn}	16.54 ± 0.77^{klm}
JG28	75.75 ± 1.07^{jklmno}	3.3 ± 0.04^{klmn}	242.84 ± 3.83^{n}	207.83 ± 5.44^{jklmn}	19.98 ± 0.47^{ijklmn}	0.33 ± 0.01^{ijkl}	15.32 ± 0.73^{fghijkl}
JG32	74.45 ± 1.02^{hijklm}	3.2 ± 0.02^{ij}	238.78 ± 1.72^{mn}	191.08 ± 2.9^{ghi}	18.71 ± 0.4^{fghij}	0.31 ± 0.01^{fghi}	14.47 ± 0.72^{defghij}
JG33	71.43 ± 2.2^{defgh}	3.89 ± 0.04^{t}	253 ± 2.68^{pq}	176.17 ± 11.8^{bcdef}	17.46 ± 0.78^{cdef}	0.28 ± 0.02^{bcdef}	13.58 ± 1.88^{cdefg}
JG36	75.01 ± 0.25^{ijklmn}	3.14 ± 0.03^{hi}	147.17 ± 1.63^{b}	219.81 ± 1.86^{no}	20.03 ± 0.32^{jklmn}	0.33 ± 0.01^{jklm}	15.98 ± 0.6^{hijklm}
JG42	74.9 ± 0.76^{hijklmn}	2.94 ± 0.02^{ef}	176.83 ± 2.88^{d}	197.08 ± 3.91^{hijkl}	18.58 ± 0.42^{fgh}	0.31 ± 0.01^{fghi}	15.29 ± 0.81^{fghijkl}
JG6	75.99 ± 1.8^{lmnop}	3.62 ± 0.03^{p}	148.88 ± 2.46^{b}	199.45 ± 2.26^{hijkl}	18.94 ± 0.5^{ghijk}	0.31 ± 0.01^{ghij}	14.75 ± 1.01^{fghijkl}
JG63	78.83 ± 2.3^{op}	3.66 ± 0.02^{pqr}	115.42 ± 1.74^{a}	217.15 ± 14.12^{mno}	20.85 ± 1.37^{no}	0.34 ± 0.02^{lmn}	16.74 ± 1.58^{lm}
JG74	77.59 ± 2.19^{mnop}	2.65 ± 0.02^{ab}	208.43 ± 4.12^{h}	209.09 ± 3.82^{klmn}	20.27 ± 0.86^{klmno}	0.34 ± 0.02^{jklm}	15.71 ± 1.03^{hijklm}
PG205	78.81 ± 2.22^{op}	2.7 ± 0.03^{bc}	244.23 ± 3.41^{no}	224.58 ± 3.35^{o}	22.31 ± 0.35^{pq}	0.37 ± 0.01^{no}	16.32 ± 0.67^{jklm}
**Mean**	**73.48±4.77**	**3.26±0.37**	**207.48±34.42**	**194.22±20.39**	**18.92±2.05**	**0.31±0.04**	**14.22±2.2**

Data pooled for two successive seasons and presented as mean of triplicate ± standard deviation. Means with the same letter are not significantly different at 5% level. Where RWC, CTD, SLA, Ci, Pn, gs, Tr indicates for relative water content, canopy temperature depression, specific leaf area, internal CO_2 concentration, photosynthetic rate, stomatal conductance, and transpiration rate, respectively.

Supplementary Material Table S3. Physiological responses of various chickpea genotypes under terminal drought stressed condition.

Genotypes	RWC	CTD	SLA	Ci	Pn	gs	Tr
ICC4958	74.23±1.96^{nopqr}	2.11±0st	136.79±1.64^{h}	172.05±10.33^{ijkl}	14.64±0.77^{klmno}	0.25±0.02^{jklmn}	12.01±0.54^{jklmn}
ICCV15102	69.72±1.3^{jklm}	1.75±0.03^{mn}	173.42±2.22^{o}	151.11±4.37^{cdefgh}	12.98±0.41^{fghi}	0.22±0.01^{efgh}	12.15±0.56^{efgh}
ICCV15115	74.34±1.46^{opqr}	1.39±0.02^{d}	146.43±1.26^{i}	181.33±4.33^{jklmno}	15.51±0.79^{mnopq}	0.26±0.02^{lmnop}	14.07±0.46^{lmnop}
ICCV15118	72.29±2.26^{lmnop}	1.64±0.01^{hi}	169.61±2.08^{o}	164.84±2.76^{ghijklmno}	15.64±0.77^{nopq}	0.26±0.02^{lmnop}	14.01±1.1^{lmnop}
ICCV181664	72.90±1.57^{mnopq}	1.75±0.03^{mn}	181.87±2.63^{p}	182.06±6.35^{jklmno}	15.89±0.76^{opq}	0.27±0.01^{nop}	13.35±0.13^{nop}
ICCV19616	62.21±2.03^{cde}	2.18±0.01^{v}	131.21±1.66^{ef}	136.23±3.12^{abcd}	11.8±0.71^{bcdef}	0.2±0.01^{bcde}	13.51±4.93^{bcde}
JAKI9218	74.74±0.63^{pqrs}	2.03±0.01^{r}	150.83±1.58^{j}	185.57±4.8^{lmno}	15.32±0.53^{lmnopq}	0.26±0.01^{lmnop}	13.22±1.35^{lmnop}
JG11	75.88±1.06^{qrs}	2.02±0.02^{qr}	154.06±0.91^{jk}	184.7±9.9^{lmno}	15.64±0.8^{nopq}	0.26±0.02^{lmnop}	13.78±0.76^{lmnop}
JG14	64.44±1.45^{defgh}	1.14±0.01^{b}	180.22±3.33^{p}	164.92±5.23^{ghi}	13.9±0.52^{hijk}	0.24±0.01^{ghijk}	10.9±0.6^{ghijk}
JG16	73.8±0.93^{nopqr}	2±0.02^{q}	111.65±0.5^{d}	171.19±6^{ijkl}	15.51±0.41^{mnopq}	0.26±0.01^{lmnop}	13.94±0.05^{lmnop}
JG17	63.67±1.01^{defg}	2±0.01^{q}	160.6±4.35^{mn}	152.31±2.5^{efgh}	12.32±0.26^{cdefg}	0.21±0.01^{cdef}	11.01±0.58^{cdef}
JG2003-14-16	72.37±2.37^{lmnop}	1.61±0.02^{h}	189.74±4.26^{qr}	162.44±8.83^{fghi}	14.79±1^{klmnop}	0.25±0.02^{jklmn}	12.23±1.1^{jklmn}
JG2016-1411	73.06±1.94^{mnopq}	1.63±0.01^{hi}	101.14±0.46^{b}	181.46±9.02^{jklmno}	16.02±0.66^{pq}	0.27±0.01^{op}	12.71±0.45^{op}
JG2016-1614	58.64±2.43^{ab}	1.58±0.03^{g}	142.61±2.93^{i}	127.6±8.97^{a}	11.29±0.75^{abc}	0.19±0.01^{abc}	10.08±1.76^{abc}
JG2016-36	57.03±1.03^{a}	1.46±0.01^{e}	173.31±4.4^{o}	128.13±5.13^{a}	11.18±0.24^{abc}	0.19±0.01^{abc}	8.71±0.6^{abc}
JG2016-44	69.42±2.26^{jkl}	1.51±0.03^{f}	130.3±1.22^{ef}	193.38±5.14^{no}	18.31±1.16^{r}	0.31±0.02^{q}	15.4±0.93^{q}
JG2016-45	62.91±1.62^{cdef}	1.76±0.01^{mn}	155.47±2^{kl}	127.74±5.28^{a}	11.33±0.32^{abc}	0.19±0.01^{abc}	9.75±0.37^{abc}
JG2016-634958	66.85±4.46^{ghij}	1.68±0.01^{jk}	156.61±2.55^{klm}	171.36±20.6^{ijkl}	14.87±1.64^{klmnop}	0.25±0.03^{jklmn}	11.92±1.55^{jklmn}
JG2016-74315	64.84±1.95^{efgh}	1.91±0.02^{p}	163.97±2.54^{n}	145.52±5.02^{bcde}	11.91±0.72^{bcdefg}	0.2±0.01^{bcde}	9.81±0.24^{bcde}
JG2016-921814	65.58±1.65^{fghi}	1.65±0.03^{ij}	172.5±4.01^{o}	151.62±4.95^{defgh}	13.72±0.48^{hijk}	0.23±0.01^{ghij}	12.73±0.88^{ghij}
JG2016-9605	57.63±1.19^{a}	1.09±0.02^{a}	186.16±2.69^{q}	136.04±4.22^{abc}	11.01±0.29^{ab}	0.18±0.01^{abc}	9.21±1.16^{ab}
JG2016-9651	62.96±0.89^{cdef}	1.77±0.01^{no}	163.42±2.52^{n}	147.73±5.88^{cdef}	12.68±0.13^{defgh}	0.22±0.01^{defg}	10.99±0.84^{defg}
JG2017-48	62.36±1.42^{cdef}	2.13±0.01^{tu}	159.67±2.76^{lmn}	128.18±13.97^{a}	11.48±0.43^{abcd}	0.19±0.01^{abc}	9.67±0.84^{abc}
JG2018-51	67.38±1.45^{hij}	2.14±0.01^{u}	179.16±0.33^{p}	151.55±11.53^{bcdeghi}	13.13±0.58^{ghij}	0.22±0.01^{efgh}	11.85±2.15^{efgh}
JG2021-1424	62.22±1.44^{cde}	1.9±0.01^{p}	191.23±1.67^{r}	151.54±4.16^{defgh}	12.91±0.16^{fgh}	0.22±0.01^{efgh}	10.37±0.72^{efgh}
JG2021-1617	59.80±1.81^{abc}	1.8±0.03^{o}	174.04±1.49^{o}	139.19±6.84^{abcde}	11.61±0.66^{bcde}	0.2±0.01^{bcd}	9.26±0.67^{bcd}
JG2021-6301	61.44±2.3^{bcd}	1.75±0.03^{mn}	135.81±3.19^{gh}	149.71±16^{cdefg}	12.77±0.72^{efgh}	0.22±0.01^{defg}	11.04±1.52^{defg}
JG2022-74	62.04±0.84^{cde}	1.44±0.02^{e}	132.16±2.49^{fg}	132.29±6.49^{ab}	11.47±0.28^{abcd}	0.19±0.01^{abc}	9.58±1.04^{abc}
JG2022-75	58.72±0.76^{ab}	1.24±0.01^{c}	172.62±3.08^{o}	123.78±8.5^{a}	10.31±0.28^{a}	0.17±0.01^{a}	8.62±1.67^{a}

JG226	70.93±2.73^{klmn}	1.11±0.02^{ab}	127.25±1.23^{e}	182.82±8.98^{klmno}	15.88±0.93^{opq}	0.27±0.02^{mnop}	13.57±0.83^{mnop}
JG24	77±0.99^{rs}	1.71±0.05^{kl}	187.53±4.46^{qr}	189.68±4.93^{mno}	16.51±0.68^{q}	0.28±0.01^{p}	13.76±0.47^{p}
JG28	68.50±0.89^{ijk}	1.71±0.02^{l}	180.77±3.55^{p}	162.81±12.27^{fghi}	14.52±0.39^{klmn}	0.24±0.01^{ijklm}	12.1±0.79^{ijklm}
JG32	71.41±1k^{lmnop}	1.66±0.02^{ij}	172.82±2.49^{o}	168.88±3.34^{ijk}	14.7±0.32^{klmno}	0.25±0.01^{jklmn}	11.98±0.21^{jklmn}
JG33	67.55±2.42^{hij}	1.75±0.02^{mn}	190.43±2.26^{qr}	152.62±9.49^{efgh}	13.07±0.81^{ghij}	0.22±0.02^{fghi}	11.9±0.47^{fghi}
JG36	67.16±0.58^{hij}	1.58±0.03^{g}	107.51±1.56^{c}	183.94±3.43^{klmno}	14.48±0.27^{klmn}	0.26±0.03^{klmno}	12.58±0.94^{klmno}
JG42	71.31±0.87^{klmno}	1.4±0.01^{d}	132.01±2.6^{fg}	164.09±4.84^{ghi}	14.15±0.41^{ijkl}	0.24±0.01^{hijkl}	12.68±0.9^{hijkl}
JG6	69.72±1.51^{jklm}	1.08±0.02^{a}	106.18±2.3^{c}	166.63±3.2^{hij}	14.28±0.5^{jklm}	0.24±0.01^{ijklm}	11.76±0.93^{ijklm}
JG63	77.66±2.51^{s}	2.08±0.01^{s}	90.68±1.82^{a}	178.13±5.08^{ijklmn}	16.39±1.12^{q}	0.28±0.02^{p}	14.61±2.49^{p}
JG74	69.09±2.23^{jkl}	1.09±0.01^{a}	141.98±1.81^{i}	176.48±12.38^{ijklm}	15.46±0.75^{mnopq}	0.26±0.01^{lmnop}	12.59±0.55^{lmnop}
PG205	77.60±0.84^{s}	1.74±0.02^{lm}	146.01±1.54^{i}	195.9±6.77^{o}	17.92±0.39^{r}	0.3±0.01^{q}	13.3±0.32^{q}
**Mean**	**67.78±6.03**	**1.67±0.31**	**153.99±26.89**	**160.44±21.67**	**13.93±2.06**	**0.24±0.04**	**11.92±2.01**

Data pooled for two successive seasons and presented as mean of triplicate ± standard deviation. Means with the same letter are not significantly different at 5% level. Where RWC, CTD, SLA, Ci, Pn, gs, Tr indicates for relative water content, canopy temperature depression, specific leaf area, internal CO_2 concentration, photosynthetic rate, stomatal conductance, and transpiration rate, respectively.

Supplementary Material Table S4. Pooled biochemical responses of various chickpea genotypes under normal irrigated condition.

**Genotypes**	**Chla**	**Chlb**	**Protein**	**H_2O_2**	**EL (%)**	**MDA**	**Sugar**	**Proline**
ICC4958	0.47±0.01[defg]	0.42±0.03[q]	0.54±0.04[c]	1.51±0.1[b]	29.65±1.36[abcd]	5.55±0.14[p]	1.43±0.11[u]	28.55±1.2[p]
ICCV15102	0.47±0.03[efgh]	0.37±0.03[ij]	0.57±0.04[g]	1.62±0.08[efg]	34.19±2.19[klm]	7.47±0.1[xy]	1.27±0.1[mn]	32.82±1.13[u]
ICCV15115	0.47±0.02[defg]	0.4±0.02[o]	0.57±0.03[gh]	1.68±0.07[jk]	32.18±1.46[hij]	2.08±0.1[c]	1.23±0.1[jkl]	20.93±1.27[i]
ICCV15118	0.47±0.03[def]	0.37±0.02[ijk]	0.57±0.03[gh]	1.63±0.06[fghi]	33.08±1.37[ijk]	4.85±0.12[mn]	1.27±0.1[n]	20.99±1.12[i]
ICCV181664	0.46±0.02[cd]	0.36±0.04[i]	0.59±0.06[k]	1.76±0.08[m]	34.47±1.56[klm]	7.38±0.13[x]	1.24±0.1[kl]	28.94±1.23[pq]
ICCV19616	0.48±0.02[gh]	0.34±0.02[fgh]	0.57±0.03[gh]	1.66±0.07[ij]	33.63±2[jklm]	7.02±0.08[w]	1.15±0.09[de]	33.84±1.01[v]
JAKI9218	0.48±0.02[gh]	0.40±0.03[op]	0.52±0.04[a]	1.41±0.1[a]	30.30±1.12[abcdefg]	6.94±0.1[w]	1.40±0.11[t]	29.88±1.01[r]
JG11	0.48±0.03[gh]	0.39±0.03[no]	0.55±0.04[de]	1.55±0.09[c]	29.81±1.26[abcde]	6.76±0.32[v]	1.41±0.1[t]	27.91±1.12[o]
JG14	0.47±0.03[efgh]	0.26±0.03[c]	0.58±0.04[j]	1.76±0.08[m]	31.77±1.99[ghi]	4.27±0.17[j]	1.22±0.1[ijk]	21.96±1.55[j]
JG16	0.50±0.03[i]	0.35±0.03[gh]	0.54±0.03[c]	1.51±0.07[b]	30.35±1.53[abcdefg]	7.88±0.35[z]	1.19±0.09[g]	32.17±1.34[t]
JG17	0.50±0.03[i]	0.23±0.02[b]	0.56±0.03[f]	1.62±0.12[fgh]	31.72±1.37[ghi]	4.50±0.08[k]	1.29±0.12[o]	19.47±1.23[de]
JG2003-14-16	0.44±0.03[b]	0.34±0.03[efg]	0.58±0.04[hi]	1.71±0.08[kl]	32.07±2.15[hij]	3.71±0.1[h]	1.18±0.1[fg]	28.82±1.3[p]
JG2016-1411	0.48±0.03[fgh]	0.34±0.03[gh]	0.57±0.05[gh]	1.69±0.06[kl]	32.12±1.84[hij]	1.32±0.14[a]	1.28±0.11[no]	17.18±1.12[a]
JG2016-1614	0.46±0.03[cde]	0.42±0.02[q]	0.57±0.03[gh]	1.71±0.08[kl]	30.79±1.17[cdefgh]	3.62±0.11[h]	1.21±0.09[hi]	19.09±1.18[cd]
JG2016-36	0.47±0.03[def]	0.40±0.02[o]	0.61±0.04[m]	1.88±0.08[o]	35.01±2.31[m]	4.23±0.08[j]	1.26±0.09[mn]	17.67±1.01[b]
JG2016-44	0.47±0.03[def]	0.38±0.03[jklm]	0.59±0.03[k]	1.75±0.1[m]	33.20±1.74[ijkl]	7.60±0.08[y]	1.22±0.09[ij]	34.22±1.01[v]
JG2016-45	0.48±0.03[h]	0.37±0.03[jkl]	0.57±0.03[gh]	1.64±0.07[ghi]	32.08±2.57[hij]	4.96±0.22[n]	1.24±0.1[jkl]	27.41±1.23[n]
JG2016-634958	0.48±0.03[h]	0.33±0.04[e]	0.59±0.03[k]	1.75±0.07[m]	29.13±1.86[ab]	6.11±0.07[t]	1.47±0.11[v]	22.25±1.01[j]
JG2016-74315	0.48±0.02[gh]	0.40±0.02[o]	0.57±0.04[gh]	1.65±0.08[hij]	30.77±2.24[cdefgh]	3.41±0.17[g]	1.17±0.1[ef]	23.97±1.01[k]
JG2016-921814	0.46±0.02[cd]	0.42±0.03[q]	0.57±0.03[gh]	1.62±0.12[fgh]	28.87±1.55[a]	4.90±0.08[n]	1.43±0.1[u]	27.53±1.12[o]
JG2016-9605	0.49±0.03[i]	0.39±0.04[mn]	0.56±0.03[f]	1.63±0.08[fghi]	30.45±2.16[abcdefg]	4.60±0.28[kl]	1.04±0.09[a]	41.81±1.95[w]
JG2016-9651	0.47±0.02[defg]	0.34±0.02[fgh]	0.64±0.03[o]	1.97±0.08[q]	30.37±1.7[abcdefg]	4.05±0.09[i]	1.32±0.1[p]	19.72±1.01[ef]
JG2017-48	0.47±0.03[efgh]	0.37±0.03[ij]	0.60±0.03[l]	1.79±0.1[n]	33.44±1.17[jklm]	5.84±0.09[qr]	1.46±0.1[v]	28.87±1.12[pq]
JG2018-51	0.48±0.03[fgh]	0.35±0.03[gh]	0.60±0.03[l]	1.87±0.07[o]	33.51±1.56[jklm]	5.24±0.08[o]	1.35±0.1[rs]	22.25±1.01[j]
JG2021-1424	0.46±0.02[cd]	0.41±0.03[pq]	0.65±0.03[p]	2.03±0.09[r]	30.03±1.42[abcdef]	2.97±0.12[f]	1.19±0.1[gh]	17.61±1.3[ab]
JG2021-1617	0.46±0.02[cd]	0.37±0.03[ij]	0.63±0.03[n]	1.98±0.09[q]	28.94±1.55[a]	7.37±0.07[x]	1.23±0.09[jk]	31.22±1.01[s]
JG2021-6301	0.47±0.02[defg]	0.40±0.04[op]	0.69±0.04[q]	2.22±0.08[s]	29.47±1.48[abc]	2.6±0.14[e]	1.47±0.12[v]	19.96±1.27[fg]
JG2022-74	0.46±0.03[cde]	0.40±0.03[op]	0.56±0.03[f]	1.59±0.08[de]	30.27±2.6[abcdefg]	2.87±0.11[f]	1.14±0.09[cd]	18.7±1.27[c]
JG2022-75	0.47±0.03[efgh]	0.35±0.03[h]	0.63±0.03[n]	1.94±0.07[p]	33.94±3.02[klm]	6.18±0.13[t]	1.09±0.09[b]	25.36±1.3[m]

JG226	0.40±0.05^{a}	0.40±0.02^{o}	0.58±0.04^{j}	1.71±0.06^{kl}	34.58±1.47^{klm}	2.26±0.08^{d}	1.25±0.09^{lm}	20.29±1.01^{gh}
JG24	0.48±0.03^{h}	0.28±0.03^{d}	0.56±0.04^{ef}	1.56±0.08^{cd}	31.12±1.65^{defgh}	4.53±0.22^{k}	1.16±0.09^{e}	19.21±1.68^{d}
JG28	0.50±0.02^{i}	0.21±0.03^{a}	0.57±0.04^{g}	1.61±0.09^{ef}	31.16±1.66^{defgh}	4.22±0.11^{j}	1.13±0.1^{c}	21.95±1.23^{j}
JG32	0.48±0.03^{h}	0.33±0.02^{ef}	0.57±0.03^{gh}	1.65±0.08^{hij}	31.02±1.72^{cdefgh}	5.21±0.12^{o}	1.33±0.1^{pq}	24.74±1.23^{l}
JG33	0.48±0.03^{fgh}	0.34±0.03^{efg}	0.57±0.04^{gh}	1.71±0.08^{kl}	31.34±1.33^{efgh}	1.73±0.09^{b}	1.36±0.1^{s}	20.61±1.12^{hi}
JG36	0.45±0.03^{c}	0.38±0.03^{lmn}	0.58±0.03^{ij}	1.71±0.07^{kl}	34.09±1.52^{klm}	6.07±0.16st	1.13±0.1^{c}	32.55±1.49^{tu}
JG42	0.48±0.02^{gh}	0.41±0.03^{pq}	0.58±0.04^{j}	1.72±0.09^{l}	34.71±1.73^{lm}	6.46±0.1^{u}	1.15±0.09^{de}	32.76±1.24^{u}
JG6	0.47±0.03^{efgh}	0.35±0.03^{gh}	0.54±0.03^{c}	1.49±0.07^{b}	31.65±2.21^{fghi}	5.75±0.09^{q}	1.26±0.12^{mn}	29.31±1.01^{q}
JG63	0.47±0.03^{defg}	0.37±0.02^{ijk}	0.53±0.04^{b}	1.48±0.06^{b}	30.64±1.39^{bcdefgh}	4.50±0.24^{k}	1.44±0.1^{u}	27.22±1.3^{n}
JG74	0.46±0.03^{cde}	0.38±0.02^{klm}	0.55±0.03^{d}	1.57±0.08^{cd}	30.35±1.55^{abcdefg}	5.93±0.11^{rs}	1.34±0.1^{qr}	29.34±1.2^{q}
PG205	0.46±0.02^{cd}	0.35±0.03^{gh}	0.57±0.03^{gh}	1.65±0.08^{ghi}	34.67±1.63^{klm}	4.72±0.08^{lm}	1.09±0.09^{b}	18±1.01^{b}
**Mean**	**0.47±0.04**	**0.36±0.07**	**0.58±0.06**	**1.7±0.22**	**31.77±2.94**	**4.94±1.77**	**1.26±0.2**	**25.43±6.89**

Data pooled for two successive seasons and presented as mean of triplicate ± standard deviation. Means with the same letter are not significantly different at 5% level. Where, Chla, Chlb, H_2O_2, EL and MDA indicate chlorophyll a, chlorophyll b, hydrogen peroxide, electrolyte leakage and malondialdehyde, respectively.

Supplementary Material Table S5. Biochemical responses of various chickpea genotypes under terminal drought stressed condition.

**Genotypes**	**Chla**	**Chlb**	**Protein**	**H_2O_2**	**EL (%)**	**MDA**	**Sugar**	**Proline**
ICC4958	0.39±0.02^{j}	0.31±0.02^{r}	0.38±0.05^{ijk}	3.6±0.08^{ij}	35±1.48^{a}	6.63±0.37^{hi}	2.07±0.12^{x}	86.17±1.66^{q}
ICCV15102	0.38±0.03^{hi}	0.24±0.01^{kl}	0.36±0.07^{cd}	3.51±0.09^{cd}	34.52±3.66^{a}	9.95±0.28^{o}	1.84±0.11^{no}	69.51±1.3^{n}
ICCV15115	0.36±0.03^{de}	0.26±0.03^{no}	0.38±0.05^{ijk}	3.53±0.11^{defg}	40.47±3.3^{cdef}	2.89±0.19^{b}	1.83±0.15^{klmno}	61.64±1.9^{hijk}
ICCV15118	0.37±0.01^{fg}	0.25±0.03^{mno}	0.39±0.05^{lmn}	3.8±0.08^{pq}	40.04±1.94^{bcdef}	6.72±0.11^{hi}	1.81±0.11^{ijklm}	60.58±1.13^{fgh}
ICCV181664	0.38±0.03^{hi}	0.24±0.02^{kl}	0.40±0.04^{n}	3.6±0.07^{j}	41.60±2.39^{defg}	9.47±0.13^{n}	1.84±0.11^{mno}	61.64±1.35^{hijk}
ICCV19616	0.36±0.05^{de}	0.22±0.03^{ij}	0.40±0.05^{mn}	3.54±0.09^{defg}	41.26±1.18^{cdefg}	10.25±0.25^{o}	1.74±0.11^{def}	69.1±1.37^{mn}
JAKI9218	0.39±0.02^{j}	0.31±0.04^{r}	0.36±0.05^{c}	3.41±0.07^{a}	36.45±1.28^{ab}	8.37±0.27^{kl}	2.05±0.11^{wx}	86.85±2.18^{q}
JG11	0.39±0.02^{j}	0.29±0.02^{q}	0.39±0.04^{klm}	3.46±0.08^{b}	34.49±1.49^{a}	9.42±0.33^{n}	2.06±0.11^{wx}	89.18±1.64^{r}
JG14	0.37±0.02^{fg}	0.16±0.02^{d}	0.38±0.05^{ghi}	3.72±0.08^{mn}	42.96±1.37^{efg}	5.84±0.33^{f}	1.8±0.13^{hijk}	58.9±1.44^{de}
JG16	0.41±0.03^{k}	0.24±0.05^{kl}	0.37±0.04^{efg}	3.57±0.08^{ghij}	39.04±1.33^{bcde}	10.11±0.2^{o}	1.83±0.12^{lmno}	89.09±1.56^{r}
JG17	0.39±0.02^{j}	0.13±0.03^{b}	0.38±0.08^{hij}	3.74±0.11^{no}	40.78±1.41^{cdef}	6.50±0.16^{hi}	1.85±0.13^{no}	56.51±1.13^{ab}
JG2003-14-16	0.33±0.02^{b}	0.22±0.02^{i}	0.36±0.04^{cd}	3.41±0.08^{a}	39.89±2.39^{bcdef}	5.24±0.17^{e}	1.76±0.12^{efg}	62.32±1.71^{jkl}
JG2016-1411	0.38±0.04^{i}	0.23±0.03^{k}	0.37±0.05^{fgh}	3.52±0.07^{def}	42.32±1.35^{efg}	2.05±0.16^{a}	1.83±0.12^{klmn}	56.51±1.35^{ab}
JG2016-1614	0.38±0.03^{hi}	0.25±0.02^{lmn}	0.39±0.04^{klm}	3.51±0.08^{cde}	39.66±1.91^{bcdef}	6±0.17^{f}	1.78±0.1^{ghi}	58.49±1.64^{d}
JG2016-36	0.38±0.02^{i}	0.25±0.03^{lm}	0.42±0.04^{p}	3.77±0.1^{op}	40.54±1.9^{cdef}	6.81±21.48^{i}	1.84±0.12^{no}	57.19±1.77^{bc}
JG2016-44	0.38±0.02^{i}	0.26±0.02^{op}	0.42±0.04^{p}	3.84±0.09^{rs}	42.13±1.84^{efg}	10.22±0.15^{o}	1.8±0.11^{hijkl}	67.94±1.34^{m}
JG2016-45	0.38±0.02^{i}	0.22±0.03^{hi}	0.37±0.05^{def}	3.66±0.08^{k}	41.54±1.48^{defg}	7.40±0.12^{j}	1.83±0.11^{lmno}	61.96±1.3^{ijk}
JG2016-634958	0.37±0.02^{fg}	0.21±0.02^{h}	0.40±0.05^{mn}	3.58±0.08^{hij}	45.08±1.84^{g}	9.12±0.25^{n}	2.07±0.13^{wx}	61.21±1.62^{ghijk}
JG2016-74315	0.36±0.02^{d}	0.23±0.02^{jk}	0.39±0.05^{jkl}	3.67±0.09^{kl}	40.97±1.75^{cdef}	5.18±0.09^{e}	1.74±0.1^{def}	62.16±1.17^{jk}
JG2016-921814	0.36±0.02^{d}	0.26±0.02^{op}	0.37±0.04^{efg}	3.6±0.11^{j}	43.83±1.84^{fg}	7.48±0.28^{j}	1.99±0.12^{u}	62.17±1.78^{jk}
JG2016-9605	0.38±0.04^{hi}	0.23±0.02^{jk}	0.34±0.04^{b}	3.48±0.07^{bc}	40.92±2.02^{cdef}	8.73±0.53^{m}	1.60±0.11^{a}	71.93±1.78^{o}
JG2016-9651	0.36±0.02^{d}	0.23±0.06^{jk}	0.42±0.04^{p}	3.81±0.09^{qr}	42.59±2.49^{efg}	6.38±0.64^{gh}	1.89±0.11^{pq}	56.31±1.17^{ab}
JG2017-48	0.37±0.02^{fg}	0.25±0.02^{lmn}	0.41±0.04^{o}	3.75±0.08^{no}	40.55±2.08^{cdef}	7.52±0.14^{j}	2.02±0.11^{uv}	58.9±1.41^{de}
JG2018-51	0.37±0.03^{gh}	0.24±0.02^{kl}	0.42±0.04^{p}	3.74±0.08^{no}	40.38±2.16^{bcdef}	7.53±0.14^{j}	1.96±0.11^{t}	61.48±1.4^{hijk}
JG2021-1424	0.37±0.02^{fg}	0.26±0.02^{op}	0.44±0.04^{q}	3.77±0.08^{op}	40.69±1.51^{cdef}	4.93±0.36^{e}	1.77±0.11^{fgh}	58.27±1.32^{cd}
JG2021-1617	0.37±0.02^{fg}	0.24±0.02^{kl}	0.45±0.05^{q}	4.09±0.13^{v}	39.80±1.65^{bcdef}	11.22±0.16^{q}	1.79±0.13^{hij}	63.52±2.05^{l}
JG2021-6301	0.37±0.03^{ef}	0.26±0.02^{op}	0.47±0.05^{r}	3.89±0.15^{t}	41.64±1.67^{defg}	4.16±0.42^{d}	2.04±0.11^{vw}	62.56±1.47^{kl}
JG2022-74	0.37±0.03^{ef}	0.25±0.02^{lmn}	0.40±0.04^{n}	3.7±0.08^{lm}	39.36±1.84^{bcde}	4.39±0.21^{d}	1.69±0.1^{c}	61.17±1.74^{fghijk}
JG2022-75	0.37±0.05^{fg}	0.23±0.02^{jk}	0.44±0.04^{q}	3.81±0.09^{qr}	39.02±1.65^{bcde}	8.12±0.13^{k}	1.64±0.11^{b}	55.83±1.29^{a}

JG226	0.30±0.02^{a}	0.24±0.02^{kl}	0.37±0.04^{efg}	3.56±0.08^{fghi}	42.40±1.55^{efg}	3.58±0.18^{c}	1.82±0.11^{jklmn}	61.62±2.05^{hijk}
JG24	0.38±0.02^{i}	0.15±0.02^{c}	0.36±0.04^{cd}	3.5±0.07^{cd}	42.25±1.47^{efg}	6.6±0.17^{hi}	1.72±0.11^{d}	62.09±1.58^{jk}
JG28	0.39±0.02^{j}	0.10±0.03^{a}	0.36±0.05^{cde}	3.55±0.09^{efgh}	37.19±1.44^{abc}	6.59±0.18^{hi}	1.72±0.1^{d}	62.5±1.98^{kl}
JG32	0.37±0.03^{gh}	0.18±0.02^{e}	0.36±0.04^{cd}	3.39±0.11^{a}	39.49±1.19^{bcde}	7.44±0.3^{j}	1.91±0.12^{qr}	61.86±1.54^{hijk}
JG33	0.38±0.02^{i}	0.19±0.02^{f}	0.38±0.05^{ghi}	3.76±0.08^{no}	41.81±1.34^{efg}	2.71±0.09^{b}	1.95±0.12st	60.64±2.68^{fghi}
JG36	0.37±0.04^{gh}	0.22±0.02^{i}	0.39±0.04^{klm}	3.86±0.13st	42.03±2.86^{efg}	8.37±0.23^{kl}	1.73±0.11^{de}	60.08±1.79^{ef}g
JG42	0.37±0.03^{gh}	0.24±0.02^{kl}	0.39±0.05^{lmn}	3.64±0.07^{k}	41.04±1.99^{cdefg}	8.55±0.17^{lm}	1.75±0.11^{defg}	62.08±2^{jk}
JG6	0.36±0.02^{d}	0.20±0.05^{g}	0.30±0.05^{a}	3.39±0.08^{a}	42.96±1.66^{efg}	10.67±0.23^{p}	1.86±0.14^{op}	59.88±1.31^{ef}
JG63	0.39±0.02^{j}	0.27±0.03^{p}	0.38±0.05^{ijk}	3.96±0.08^{u}	35.17±1.65^{a}	5.85±0.23^{f}	2.06±0.11^{wx}	80.25±1.98^{p}
JG74	0.35±0.02^{c}	0.20±0.02^{g}	0.34±0.04^{b}	3.53±0.07^{def}	41.80±1.8^{efg}	11.81±0.33^{r}	1.92±0.12^{rs}	57.84±1.69^{cd}
PG205	0.38±0.02^{i}	0.24±0.02^{kl}	0.39±0.04^{klm}	3.77±0.08^{op}	37.51±3.81^{abcd}	6.12±0.25^{fg}	1.67±0.12^{c}	60.98±2.53^{fghij}
**Mean**	**0.37±0.04**	**0.23±0.06**	**0.39±0.07**	**3.65±0.22**	**40.28±4.01**	**7.17±4.22**	**1.85±0.23**	**64.47±10.06**

Data pooled for two successive seasons and presented as mean of triplicate ± standard deviation. Means with the same letter are not significantly different at 5% level. Where, Chla, Chlb, H_2O_2, EL and MDA indicate chlorophyll a, chlorophyll b, hydrogen peroxide, electrolyte leakage and malondialdehyde, respectively.

Supplementary Material Table S6. Yield and yield attributing trait responses of various chickpea genotypes under normal irrigated condition.

Genotypes	DTF	DTM	NOP	SYPP	BYPP	HI	SW
ICC4958	61.50±0.87^{bcde}	112.33±2.02^{abcdef}	54.5±1.8^{bcdef}	11.79±0.31^{a}	33.58±0.46^{hijkl}	35.15±1.42^{bcde}	29.88±0.08^{s}
ICCV15102	66.49±0.02^{hijk}	117.25±1.32^{hijklmn}	62.04±0.37^{hijk}	15.26±0.21^{opqr}	33.07±1.21^{ghijk}	46.13±1.18^{nop}	25.31±0.69^{q}
ICCV15115	67.10±0.53^{ijkl}	114.42±2.08^{cdefghijk}	67.32±4.76^{klmno}	14.37±0.6^{klmnop}	35.03±0.55^{klmno}	41.03±2.04^{ijkl}	23.07±0.91^{nop}
ICCV15118	67±2.29^{ijkl}	116.25±0.9^{fghijklm}	60.41±3.21^{ghij}	14.38±0.66^{klmnop}	33.27±1.03^{ghijk}	43.2±1.02^{klmno}	21.18±1.37^{klm}
ICCV181664	70.93±1.01^{n}	118.08±0.8^{jklmn}	57.42±2.36^{defgh}	12±0.49^{ab}	31.86±1.24^{efgh}	37.82±1.86^{defghi}	21.23±0.06^{klm}
ICCV19616	68.71±1.94^{jklmn}	116.67±1.46^{ghijklm}	67.97±4.34^{lmnop}	12.23±0.61^{abc}	29.69±1.37^{bcd}	41.19±3.38^{ijkl}	23.48±1.34^{op}
JAKI9218	63.17±1.04^{efg}	112.83±0.29^{abcdefg}	77.58±2.75^{qr}	16.1±1.53^{qr}	34.55±1^{jklmn}	46.50±3.06^{op}	28.76±1.18^{s}
JG11	60±2.6^{abcd}	109.92±1.66^{a}	71±2.46^{nop}	15.44±0.1^{opqr}	36.32±1.41^{nopq}	42.52±1.89^{klmn}	25.82±0.37^{q}
JG14	65.17±2.02^{fghi}	110.17±1.76^{ab}	50.75±0.5^{abc}	13.14±0.19^{bcdefghij}	32.41±2.1^{efghi}	40.68±2.82^{hijkl}	20.6±0.2^{jk}
JG16	65.83±1.04^{ghij}	116.5±0.87^{fghijklm}	73.32±3.1^{pq}	16.23±0.74^{r}	48.01±0.8^{u}	33.82±1.49^{bc}	27.15±0.17^{r}
JG17	62.83±0.58^{def}	111.75±5.68^{abcde}	47.86±2.81^{a}	13.6±0.73^{efghijkl}	30.74±1.32^{cde}	44.14±1.19^{lmno}	20.21±0.03^{ijk}
JG2003-14-16	58.50±1.73^{a}	109.92±3.19^{a}	70.8±4.37^{nop}	14.87±0.13^{mnop}	40.55±1.19^{r}	36.67±0.81^{cdefg}	23.72±0.97^{p}
JG2016-1411	61.21±1.48^{abcde}	113.25±1.52^{abcdefgh}	59.73±4.94^{fghi}	13.35±0.12^{cdefghijk}	33.62±0.79^{hijkl}	39.74±0.96^{fghijk}	18.45±0.82^{defg}
JG2016-1614	67.32±2.01^{ijklm}	110.75±1.64^{abc}	52.58±0.76^{abcde}	12.47±0.8^{abcde}	36.56±0.97^{opq}	34.27±3.19^{bcd}	15.65±0.83^{b}
JG2016-36	67.33±0.76^{ijklm}	117.75±1.52^{ijklmn}	71.87±1.15^{op}	14.06±0.77^{ijklmn}	32.17±1.18^{efghi}	43.68±2.35^{klmno}	17.04±0.15^{c}
JG2016-44	67.38±0.82^{ijklm}	114±1.39^{abcdefghij}	57.79±2.51^{efgh}	13.78±0.23^{ghijklm}	37.26±0.37^{q}	36.90±0.86^{cdefgh}	20.17±0.33^{ijk}
JG2016-45	65.27±0.75^{fghi}	113.75±4.02^{abcdefghi}	59.65±3.72^{fghi}	14.01±0.87^{hijklmn}	33.05±0.43^{ghijk}	42.40±3.14^{klmn}	20.02±0.41^{hijk}
JG2016-634958	62.43±0.93^{cdef}	119.92±1.51^{mn}	61.01±4.11^{hij}	13.35±0.1^{cdefghijk}	32.28±0.71^{efghi}	41.34±0.64^{ijkl}	23.7±0.62^{p}
JG2016-74315	67.65±0.78^{ijklm}	116.5±1.25^{fghijklm}	63.08±3.01^{hijkl}	12.31±0.86^{abcd}	34.63±0.24^{jklmno}	35.61±2.6^{bcde}	18.97±0.9^{fghi}
JG2016-921814	63.99±3.12^{efgh}	111.25±1.75^{abcd}	51.92±4.01^{abcd}	11.85±0.46^{a}	30.8±1.03^{cde}	38.44±0.23^{efghij}	22.58±0.71^{nop}
JG2016-9605	64.77±0.25^{fghi}	115.17±1.44^{defghijkl}	66.75±6.26^{klmno}	13.5±0.22^{defghijk}	28.84±1.61^{b}	46.84±2.29^{op}	20.93±1.2^{jkl}
JG2016-9651	67.49±0.98^{ijklm}	113.33±1.84^{abcdefgh}	58.03±3.34^{efgh}	14.51±0.58^{klmnop}	35.77±0.73^{mnopq}	40.48±0.91^{ghijkl}	20.01±1.41^{hijk}
JG2017-48	64.88±1.32^{fghi}	114.17±1.46^{bcdefghij}	57.92±3.55^{efgh}	13.72±0.45^{fghijklm}	33.22±1.23^{ghijk}	41.34±2.13^{ijkl}	23.72±0.62^{p}
JG2018-51	69.49±1.72^{lmn}	110.92±1.38^{abc}	49.46±0.37^{abc}	14.3±0.61^{jklmno}	39.87±1.13^{r}	35.83±0.85^{bcde}	18.66±0.24^{efgh}
JG2021-1424	64±1.8^{efgh}	116.33±0.72^{fghijklm}	67.64±2.11^{klmnop}	12.93±0.76^{abcdefghi}	35.78±0.88^{mnopq}	36.12±2.08^{bcdef}	16.99±1.1^{c}
JG2021-1617	58.83±0.29^{ab}	116.25±2.63^{fghijklm}	51.42±3.75^{abc}	11.76±0.42^{a}	31.32±0.09^{defg}	37.52±1.42^{cdefghi}	18.06±0.44^{cdef}
JG2021-6301	68.67±0.76^{klm}	119.33±1.28^{lmn}	55.08±2.08^{cdefg}	12.78±0.62^{abcdefg}	37.01±0.39^{pq}	34.47±1.45^{bcd}	14.37±0.83^{a}
JG2022-74	69.65±1.24^{lmn}	117±2.61^{ghijklm}	54.06±2.03^{bcdef}	12.53±0.3^{abcdef}	31.05±0.6^{def}	40.29±0.83^{ghijkl}	20.15±0.89^{ijk}
JG2022-75	70.21±1.33^{mn}	116.75±1.98^{ghijklm}	54.75±0^{bcdefg}	12.21±0.74^{abc}	35.28±1.39^{lmnop}	34.69±3.17^{bcde}	19.07±0.66^{fghi}

JG226	63.17±1.61^{efg}	113.17±2.27^{abcdefgh}	68.06±2.48^{lmnop}	15.05±0.57^{nopq}	34.94±1.19^{klmno}	43.08±2.63^{klmno}	21.98±1.06^{lmn}
JG24	71.17±0.58^{n}	121.33±1.61^{n}	49.25±2.05^{ab}	12.26±0.32^{abc}	34.06±1.79^{ijklm}	35.96±1.74^{bcdef}	31.4±0.87^{t}
JG28	66.38±1.27^{hijk}	117.42±1.28^{hijklmn}	49.75±2.18^{abc}	11.73±0.72^{a}	25.47±0.29^{a}	45.87±2.37^{mnop}	18.94±0.26^{fghi}
JG32	78.50±1.73^{o}	115.25±4.02^{defghijkl}	69.42±3.79^{mnop}	12.56±0.24^{abcdef}	29.11±1b^{c}	43.19±0.61^{klmno}	17.08±0.38^{cd}
JG33	59.83±1.53^{abc}	115.75±1.39^{efghijklm}	65.08±2.25^{ijklm}	14.4±0.34^{klmnop}	32.83±1.17^{fghij}	43.82±2.24^{lmno}	18.17±0.3^{cdefg}
JG36	67.67±0.76^{jklmn}	118.08±1.46^{jklmn}	65.42±2.32^{jklmn}	12.84±0.81^{abcdefgh}	42.57±1.17^{s}	30.15±1.13^{a}	17.17±0.76^{cd}
JG42	62.65±1.02^{cdef}	119±3.7^{lmn}	72.25±1.8^{op}	13.11±0.4^{bcdefghij}	28.38±0.82^{b}	46.17±2.33^{nop}	19.55±0.72^{ghij}
JG6	61.67±1.15^{cde}	110.83±1.38^{abc}	62.58±2.47^{hijkl}	21.74±0.78^{s}	42.65±1.1^{s}	50.90±1.43^{q}	31.45±0.26^{t}
JG63	69.17±0.76^{klmn}	118.5±1.8^{klmn}	63.85±0.96^{ijklm}	15.51±0.97^{pqr}	32.35±0.38^{efghi}	47.98±3.17^{pq}	18.1±0.33^{cdef}
JG74	70.15±2.91^{mn}	125.33±2.02^{o}	77.75±3.04^{qr}	14.06±0.82^{hijklmn}	33.42±0.69^{hijkl}	42.06±3.31^{jklm}	17.49±0.72^{cde}
PG205	70.82±3.49^{n}	117.25±1.89^{hijklmn}	79.75±2.77^{r}	14.77±0.54^{lmnop}	45.55±0.22^{t}	32.35±1.39^{ab}	22.31±0.39^{mno}
**Mean**	**65.97±4.2**	**115.36±3.8**	**61.92±8.94**	**13.77±1.85**	**34.47±4.62**	**40.26±5.02**	**21.32±4.16**

Data pooled for two successive seasons and presented as mean of triplicate ± standard deviation. Means with the same letter are not significantly different at 5% level. Where, DTF, DTM, NOP, SYPP, BYPP, HI and SW indicate for days to flowering, days to maturity, number of pods, seed yield per plant, biological yield per plant, harvest index and hundred seed weight, respectively.

Supplementary Material Table S7. Yield and yield attributing trait responses of various chickpea genotypes under terminal drought stressed condition.

**Genotypes**	**DTF**	**DTM**	**NOP**	**SYPP**	**BYPP**	**HI**	**SW**
ICC4958	54.73±2.48^{ab}	98.13±2.31^{a}	52±0.5^{jkl}	9.2±0.36^{jklmno}	28.38±1.16^{hijk}	32.51±2.37^{abcdefgh}	29.82±0.28^{r}
ICCV15102	61.74±1.51^{efghijkl}	111.15±0.69^{ijkl}	51.4±1.95^{ijkl}	9.95±0.36^{op}	25.78±1.02^{defgh}	38.65±0.31^{klmno}	23.45±0.39^{o}
ICCV15115	61.46±0.29^{efghijk}	108.52±2.01^{efghij}	52.08±1.76^{kl}	9.7±0.32^{nop}	27.84±0.48^{hij}	35±1.69^{efghijkl}	22.39±1.01^{mno}
ICCV15118	62.65±2^{fghijklm}	108.69±0.65^{efghij}	52.5±5.94^{klm}	9.54±0.58^{lmno}	23.16±0.91^{bcd}	41.15±0.89^{nop}	20.12±1.49^{ijk}
ICCV181664	65.68±1.26^{m}	111.15±0.72^{ijkl}	42.58±4.46^{cde}	8.61±0.25^{fghij}	24.35±0.45^{bcdef}	35.34±1.38^{fghijkl}	20.84±1.44^{jkl}
ICCV19616	63.07±1.87^{ghijklm}	110±0.87^{fghijk}	49.84±0.86^{hijkl}	8.36±0.33^{defgh}	22.6±0.99^{bc}	37.02±0.35^{hijklmn}	23.35±0.85^{o}
JAKI9218	56.8±3.62^{abcd}	98.83±1.38^{a}	63.42±2.75^{pq}	11.25±0.45^{r}	26.04±0.84^{efgh}	43.29±2.29^{p}	28.08±0.4^{q}
JG11	54.30±1.61^{a}	99.5±1.32^{a}	57.58±2.47^{mno}	11.42±0.17^{r}	27.38±0.71^{ghi}	41.91±1.61^{op}	24.83±0.84^{p}
JG14	59.80±1.04^{defgh}	103.63±1.3^{b}	30.25±3.14^{a}	8.81±0.14^{fghjikl}	24.1±1.75^{bcdef}	36.68±2.78^{ghijklmn}	19.85±0.46^{hij}
JG16	58.96±2.08^{cdef}	104.67±0.58^{bc}	65.25±2.6^{q}	10.29±0.55^{pq}	33.15±1.19^{mn}	31.18±2.82^{abcdef}	26.08±0.4^{p}
JG17	58.30±0.29^{bcde}	108.06±2.49^{defghi}	34.94±2.15^{b}	8.99±0.17^{hijklmn}	24.81±4^{cdefg}	37.01±6^{hijklmn}	16.67±0.56^{cde}
JG2003-14-16	55.33±1.89^{ab}	103.52±1.22^{b}	59.53±1.98^{op}	9.56±0.08^{mno}	30.52±0.99^{kl}	31.33±1.03^{abcdef}	22.96±1.14^{no}
JG2016-1411	56.91±0.95^{abcd}	106.94±2.01^{cdef}	50.4±4.07^{ijkl}	9.01±0.3^{hijklmn}	24.18±0.61^{bcdef}	37.3±0.84^{ijklmno}	16.63±0.55^{cde}
JG2016-1614	62.96±2.02^{ghijklm}	106.07±1.49^{bcde}	45.92±1.44^{defghi}	7.79±0.12^{bcd}	24.02±1.1^{bcdef}	32.59±1.63^{abcdefghi}	14.96±1.24^{ab}
JG2016-36	63.21±0.46^{hijklm}	111.02±0.78^{ijkl}	49.69±0.59^{ghijkl}	9.16±0.63^{jklmno}	22.44±0.06^{bc}	40.91±2.76^{mnop}	16.14±0.45^{bc}
JG2016-44	63.07±0.92^{ghijklm}	107.88±0.6^{defgh}	46.36±1.06^{defghij}	8.99±0.39^{hijklmn}	25.91±0.64^{efgh}	34.76±0.74^{defghijk}	18.69±1.16^{fghi}
JG2016-45	59.95±1.26^{defgh}	107.02±1.75^{cdef}	43.47±2.05^{cdef}	9.09±0.58i^{jklmn}	23.03±1.15^{bc}	39.63±4.13^{lmnop}	18.61±0.68^{fgh}
JG2016-634958	59.56±2.71^{defgh}	112.19±1.66^{kl}	52.42±2.93^{klm}	8.84±0.56^{fghjiklm}	25.95±1.07^{efgh}	34.2±2.73^{cdefghijk}	22.32±0.46^{mno}
JG2016-74315	61.42±0.68^{efghijk}	111±1.06^{ijklm}	54.31±1.4^{lmn}	8.37±0.16^{defghi}	24.01±1.48^{bcdef}	34.96±2.72^{efghijkl}	18.15±0.93^{fg}
JG2016-921814	61.01±2.93^{efghij}	105.19±1.08^{bcd}	45.97±0.95^{defghi}	8.56±0.62^{efghij}	24.25±0.82^{bcdef}	35.39±1.78^{fghijkl}	21.82±0.49^{lmn}
JG2016-9605	59.82±2.11^{defgh}	109.5±1.11^{fghijk}	49.79±5.18^{ghijkl}	7.18±0.39^{bc}	23.61±0.94^{bcdef}	30.47±1.38^{abcde}	20±0.67^{hijk}
JG2016-9651	62.82±1.54^{ghijklm}	108.04±1.92^{defghi}	53.21±2.31^{klm}	9.57±0.29^{mno}	30.55±0.63^{kl}	31.33±0.71^{abcdef}	19.49±1.06^{ghij}
JG2017-48	60.28±1.43^{defghi}	107.42±0.45^{cdefg}	50.69±0.77^{ijkl}	9.15±0.47^{jklmno}	25.18±0.26^{cdefg}	36.4±1.92^{ghijklm}	23.24±0.87^{o}
JG2018-51	65.41±1.44^{lm}	104.48±1.88^{bc}	44.58±2.34^{cdefgh}	9.48±0.28^{lmno}	31.81±0.29^{lm}	29.89±0.6^{abc}	17.86±0.72^{def}
JG2021-1424	59.28±1.18^{defg}	111.29±0.73^{jkl}	44.58±2.89^{cdefgh}	8.13±0.24^{def}	24.09±0.53^{bcdef}	33.79±0.9^{cdefghij}	16.57±0.91^{cd}
JG2021-1617	54.59±1.28^{a}	110.82±2.78^{hijkl}	47.58±4.25^{efghijk}	7.34±0.37^{bc}	23.45±0.06^{bcde}	31.39±1.44^{abcdef}	17.69±0.1^{def}
JG2021-6301	64.74±0.98^{jklm}	113.17±0.38^{lm}	49.19±4^{ghijkl}	8.75±0.33^{fghijk}	28.12±0.74^{hijk}	31.15±0.55^{abcdef}	13.77±1.19^{a}
JG2022-74	64.23±1.93^{jklm}	110.29±2.85^{ghijkl}	48.69±2.2^{fghijkl}	7.24±0.13^{bc}	22.63±0.2^{bc}	32.14±0.69^{abcdefg}	20.04±0.82^{hijk}
JG2022-75	64.75±0.89^{jklm}	111.02±1.47^{ijkl}	50.14±0.67^{hijkl}	7.87±0.44^{cde}	28.36±3.14^{hijk}	28.37±3.93^{a}	18.6±0.69^{fgh}
JG226	60.28±1.74^{defghi}	106±1.7^{bcde}	48.26±2.91^{fghijk}	8.62±0.18^{fghij}	28.73±1.15^{ijk}	30.06±1.64^{abcd}	21.35±0.84^{klm}

JG24	65.13±0.5^{klm}	115.23±0.78^{m}	41.93±4.99^{cd}	8.15±0.22^{defg}	24.35±1.68^{bcdef}	33.59±2.28^{bcdefghij}	30.54±0.3^{r}
JG28	62.13±1.8^{fghijklm}	110.31±0.29^{ghijkl}	43.61±3.05^{cdef}	7.88±0.08^{cde}	24.29±2.69^{bcdef}	32.77±3.7^{abcdefghi}	18.05±0.47^{efg}
JG32	74.91±1.55^{n}	109.44±2.69^{fghijk}	50.34±3.1^{ijkl}	8.66±0.29^{fghij}	23±2.63^{bc}	38.06±4.72^{jklmno}	16.02±0.65^{bc}
JG33	55.67±1.79^{abc}	108.27±0.25^{efghij}	39.19±0.42^{bc}	8.67±0.07^{fghij}	22.56±0.26^{bc}	38.62±0.87^{klmno}	14.94±0.42^{ab}
JG36	62.35±1.85^{fghijklm}	112.23±0.81^{kl}	54.26±2.79^{lmn}	9.13±0.4^{jklmno}	32.54±0.14^{lmn}	28.18±1.22^{a}	15.98±0.19^{bc}
JG42	59.90±0.25^{defgh}	112.54±2.49^{klm}	44.2±1.6^{cdefg}	8.87±0.45^{ghijklm}	21.96±0.81^{ab}	40.87±3.55^{mnop}	18.73±0.81^{fghi}
JG6	56.96±1.53^{abcd}	100.15±3.28^{a}	42.1±2.31^{cde}	9.43±0.59^{klmno}	30.1±0.83^{jkl}	31.33±1.2^{abcdef}	30.56±0.7^{r}
JG63	61.80±3.82^{efghijkl}	112.04±0.79^{kl}	58.47±3.97^{nop}	10.63±0.39^{q}	26.23±2.35^{fghi}	40.85±5.03^{mnop}	17.88±0.45^{def}
JG74	64.02±3.96^{ijklm}	119.42±1.06^{n}	41.53±4.18^{cd}	6.14±0.33^{a}	19.93±0.35^{a}	30.86±1.11^{abcdef}	15.85±0.52^{bc}
PG205	65.52±3.8^{lm}	110.94±1.76^{hijkl}	62.67±2.54^{pq}	10±0.38^{opq}	34.77±1.8^{n}	28.85±0.51^{ab}	22.31±0.31^{mno}
**Mean**	**61.14±4.22**	**108.39±4.58**	**49.12±7.57**	**8.91±1.09**	**25.95±3.59**	**34.74±4.53**	**20.38±4.31**

Data pooled for two successive seasons and presented as mean of triplicate ± standard deviation. Means with the same letter are not significantly different at 5% level. Where, DTF, DTM, NOP, SYPP, BYPP, HI and SW indicate for days to flowering, days to maturity, number of pods, seed yield per plant, biological yield per plant, harvest index and hundred seed weight, respectively.

Supplementary Material Table S8. PC Scores of studied chickpea genotypes under terminal drought stressed condition.

Row Names	Genotype	PC1	PC2	PC3
1	ICC4958	-6.50136	-2.87579	1.426453
2	JAKI9218	-6.95491	-1.55947	2.531589
3	JG6	0.736056	3.502651	4.526591
4	JG11	-7.10434	-2.39003	2.021287
5	JG14	2.071282	1.543937	1.063758
6	JG16	-6.1469	-0.80386	0.428306
7	JG17	1.694379	-0.36306	1.124941
8	JG24	-1.37694	3.774933	-0.16164
9	JG28	0.943795	1.750512	0.221353
10	JG32	0.559676	1.732257	-0.30897
11	JG33	1.039403	-0.06702	0.194738
12	JG36	0.331787	1.18546	-2.09284
13	JG42	0.027835	0.753473	-0.43298
14	JG63	-5.16651	-0.91593	-1.55699
15	JG74	3.263057	4.519903	0.573507
16	JG226	0.514748	3.562517	-0.59633
17	PG205	-4.71269	1.051679	-3.60294
18	ICCV15102	-0.86241	-0.94224	1.493535
19	ICCV15115	-1.90485	1.498351	-1.24874
20	ICCV15118	-1.1294	0.892493	-1.1424
21	ICCV19616	0.057233	-0.96645	0.723528
22	ICCV181664	-0.55892	1.784882	-0.95072
23	JG2003-14-16	-1.36355	1.091568	0.766214
24	JG2016-44	-1.33749	2.587296	-1.89926
25	JG2016-45	2.362902	-1.17754	1.555341
26	JG2016-1411	-0.91501	1.959239	-0.62536
27	JG2016-1614	3.072646	-1.78623	0.537604
28	JG2016-9605	4.610555	0.199801	3.090113
29	JG2016-9651	1.180432	-1.41177	-1.51808

30	JG2016-74315	1.566116	-1.69197	-0.36157
31	JG2016-634958	-0.08124	0.577337	-0.34197
32	JG2016-921814	0.555114	0.087732	0.536136
33	JG2017-48	1.83566	-2.49895	0.378646
34	JG2018-51	0.591004	-1.3204	-1.3054
35	JG2022-74	3.664413	-1.16636	0.131153
36	JG2016-36	3.775852	-2.86033	0.000473
37	JG2022-75	4.142539	-2.45586	-0.77306
38	JG2021-6301	2.036668	-2.71955	-2.99602
39	JG2021-1424	2.14995	-1.28625	-0.95101
40	JG2021-1617	3.333416	-2.79695	-0.45899
